# Integration of Posttranscriptional Gene Networks into Metabolic Adaptation and Biofilm Maturation in *Candida albicans*

**DOI:** 10.1371/journal.pgen.1005590

**Published:** 2015-10-16

**Authors:** Jiyoti Verma-Gaur, Yue Qu, Paul F. Harrison, Tricia L. Lo, Tara Quenault, Michael J. Dagley, Matthew Bellousoff, David R. Powell, Traude H. Beilharz, Ana Traven

**Affiliations:** 1 Infection and Immunity Program, Biomedicine Discovery Institute and the Department of Biochemistry and Molecular Biology, Monash University, Clayton, Victoria, Australia; 2 Infection and Immunity Program, Biomedicine Discovery Institute and the Department of Microbiology, Monash University, Clayton, Victoria, Australia; 3 Monash Bioinformatics Platform, Monash University, Clayton, Victoria, Australia; 4 Development and Stem Cells Program, Biomedicine Discovery Institute and the Department of Biochemistry and Molecular Biology, Monash University, Clayton, Victoria, Australia; University College Dublin, IRELAND

## Abstract

The yeast *Candida albicans* is a human commensal and opportunistic pathogen. Although both commensalism and pathogenesis depend on metabolic adaptation, the regulatory pathways that mediate metabolic processes in *C*. *albicans* are incompletely defined. For example, metabolic change is a major feature that distinguishes community growth of *C*. *albicans* in biofilms compared to suspension cultures, but how metabolic adaptation is functionally interfaced with the structural and gene regulatory changes that drive biofilm maturation remains to be fully understood. We show here that the RNA binding protein Puf3 regulates a posttranscriptional mRNA network in *C*. *albicans* that impacts on mitochondrial biogenesis, and provide the first functional data suggesting evolutionary rewiring of posttranscriptional gene regulation between the model yeast *Saccharomyces cerevisiae* and *C*. *albicans*. A proportion of the Puf3 mRNA network is differentially expressed in biofilms, and by using a mutant in the mRNA deadenylase *CCR4* (the enzyme recruited to mRNAs by Puf3 to control transcript stability) we show that posttranscriptional regulation is important for mitochondrial regulation in biofilms. Inactivation of *CCR4* or dis-regulation of mitochondrial activity led to altered biofilm structure and over-production of extracellular matrix material. The extracellular matrix is critical for antifungal resistance and immune evasion, and yet of all biofilm maturation pathways extracellular matrix biogenesis is the least understood. We propose a model in which the hypoxic biofilm environment is sensed by regulators such as Ccr4 to orchestrate metabolic adaptation, as well as the regulation of extracellular matrix production by impacting on the expression of matrix-related cell wall genes. Therefore metabolic changes in biofilms might be intimately linked to a key biofilm maturation mechanism that ultimately results in untreatable fungal disease.

## Author Summary

Metabolism is a master regulator of cell biology, including gene regulation, developmental switches and cellular life-death decisions, with the mitochondrion playing a central role in eukaryotes. For the yeast *Candida albicans* mitochondrial functions have been implicated in host-pathogen interactions, but the regulatory mechanism that control mitochondrial biogenesis are poorly described. We identified the RNA binding protein Puf3 as a new mitochondrial regulator in *C*. *albicans*, and show that posttranscriptional regulation and mitochondrial function have important roles during community growth in biofilms. Perturbation of mitochondrial activity or inactivation of a key posttranscriptional regulator, *CCR4*, led to changes in biofilm maturation, shedding light on the interface between metabolic reprogramming and biofilm developmental pathways. We illuminate a new mechanism that regulates extracellular matrix production, an essential biofilm feature that mediates the notorious drug resistance and immune evasion properties of the biofilm growth mode.

## Introduction

Metabolism is a key driver of cell growth and division, and has a widespread influence on cell function. For example, metabolites can regulate gene expression [1], metabolic enzymes can double as RNA binding proteins and regulate mRNA expression [2], and the “power house of eukaryotic cells”–the mitochondrion–plays diverse roles in nuclear gene expression control [3], as well as pathways of programmed cell death [4] and cellular aging [3,5]. We are studying mitochondrial functions in *Candida albicans*, a human commensal yeast known to cause serious infections in susceptible individuals [6]. Al Brown and colleagues have recently argued that metabolism should be put center stage for a holistic understanding of virulence and host interactions of human fungal pathogens [7]. *C*. *albicans* can inhabit several niches in the human body that differ in nutrient availability, and it has evolved sophisticated mechanisms to cope with changing nutrient environments. For example, *C*. *albicans* uses complex networks of transcriptional activators and repressors to modulate the switch from being a commensal inhabitant of the gastrointestinal (GI) tract, to becoming a pathogen localized in the blood stream [8,9]. Many of the target genes of these regulators relate to metabolic functions [8]. Like the majority of organisms, *C*. *albicans* is highly responsive to carbon source availability. Major metabolic remodeling, but also global changes in cell physiology including restructuring of the cell surface and host interactions, have been found when comparing *C*. *albicans* grown in the fermentative carbon source glucose with the non-fermentative carbon source lactate [10,11]. These carbon sources are found at varying concentrations in the GI tract, the vaginal tract and the bloodstream, and thus are relevant nutrients for *C*. *albicans* in host environments [7].

Metabolic control is linked to a critical virulence attribute of *C*. *albicans*—morphological plasticity, whereby this organism transitions between distinct cell types in response to environmental signals. One such developmental transition of central importance is substrate-attached growth of *C*. *albicans* in multicellular biofilm communities, a property that is highly relevant for virulence [12]. Biofilm formation involves several important phenotypic aspects, such as adherence, cell surface restructuring, the yeast-to-hyphae morphogenetic switch and the production of protective extracellular matrix material [13]. The pathways that drive adherence and morphogenesis have been widely studied, and several signal transduction pathways as well as a highly interconnected network of transcription factors are known to regulate biofilm formation in *C*. *albicans* [13,14]. Recent studies have begun to address the pathways required for extracellular matrix biogenesis (i.e. making the matrix components) [15,16]. However, the regulatory aspects of matrix production are poorly defined, and only two gene expression regulators are known to control matrix accumulation in biofilms: the transcription factor Rlm1 is a positive regulator [17], while the transcription factor Zap1 is a negative regulator [18].

Transcriptomics and metabolomics analyses of biofilms have revealed that a critical difference between planktonic (suspension) growth and surface-attached biofilm growth relates to metabolic reprogramming. Glycolysis, ergosterol biosynthesis, the sulfur assimilation pathway, glycerol synthesis and respiratory metabolism are all modulated in biofilms [14,19–22]. Following from these studies, deletion of differentially expressed genes required for metabolic functions in biofilms has been found to impact on biofilm formation, underscoring the importance of metabolic reprogramming for the biofilm growth mode [23,24]. Relevant to our research interests, mitochondrial function and/or biogenesis are differentially regulated in biofilms [19]. These and other studies [25,26] are consistent with a role for mitochondrial reprogramming in biofilm formation. However, the important question of how metabolic changes are superimposed onto the developmental and structural changes that drive biofilm-dependent phenotypes remains poorly elucidated.

Although mitochondrial activity is clearly important for fungal virulence [27], very little is known about the regulatory networks that control mitochondrial biogenesis in *C*. *albicans* in an environmental and/or developmental context. Three transcription factors have been shown to have a role in mitochondrial respiratory activity in this pathogen [28], and the transcriptional regulatory complex Mediator modulates respiratory metabolism, although the gene targets are not known [25]. In addition to these transcriptional mechanisms, we have previously shown that posttranscriptional regulation through the major cytoplasmic mRNA deadenylase complex Ccr4-NOT has an impact on mitochondrial biogenesis in *C*. *albicans* in planktonic cultures [29].

We report here that a proportion of the mitochondria-related genes down-regulated in mature *C*. *albicans* biofilms [14] belong to an mRNA network of putative targets of the Pumilio RNA binding protein Puf3. We performed a detailed analysis of Puf3 function in the pathogen *C*. *albicans*, and our results suggest that posttranscriptional mRNA control of genes encoding the mitochondrial ribosome has been rewired between *S*. *cerevisiae* and *C*. *albicans*. We further show that posttranscriptional gene regulation is important for mitochondrial activity in biofilms, and demonstrate that that inactivation of the mRNA deadenylase *CCR4* or uncoupling of mitochondrial oxidative phosphorylation have consequences for biofilm structure and maturation, particularly the production of the extracellular matrix. While secretion of the extracellular matrix is a property of biofilm growth that has very important consequences for biofilm resistance to antifungal drugs (reviewed in [30]), and evasion of innate immunity [31], it is the least understood of the biofilm maturation processes. Our study illuminates a new mechanism of biofilm matrix regulation, leading us to propose a model for how environmental and nutritional changes in biofilms drive a critical biofilm protection mechanism.

## Results

### The *C*. *albicans* Puf3 regulon includes mRNAs encoding mitochondrial biogenesis genes differentially expressed in biofilms

**Fig 1:**
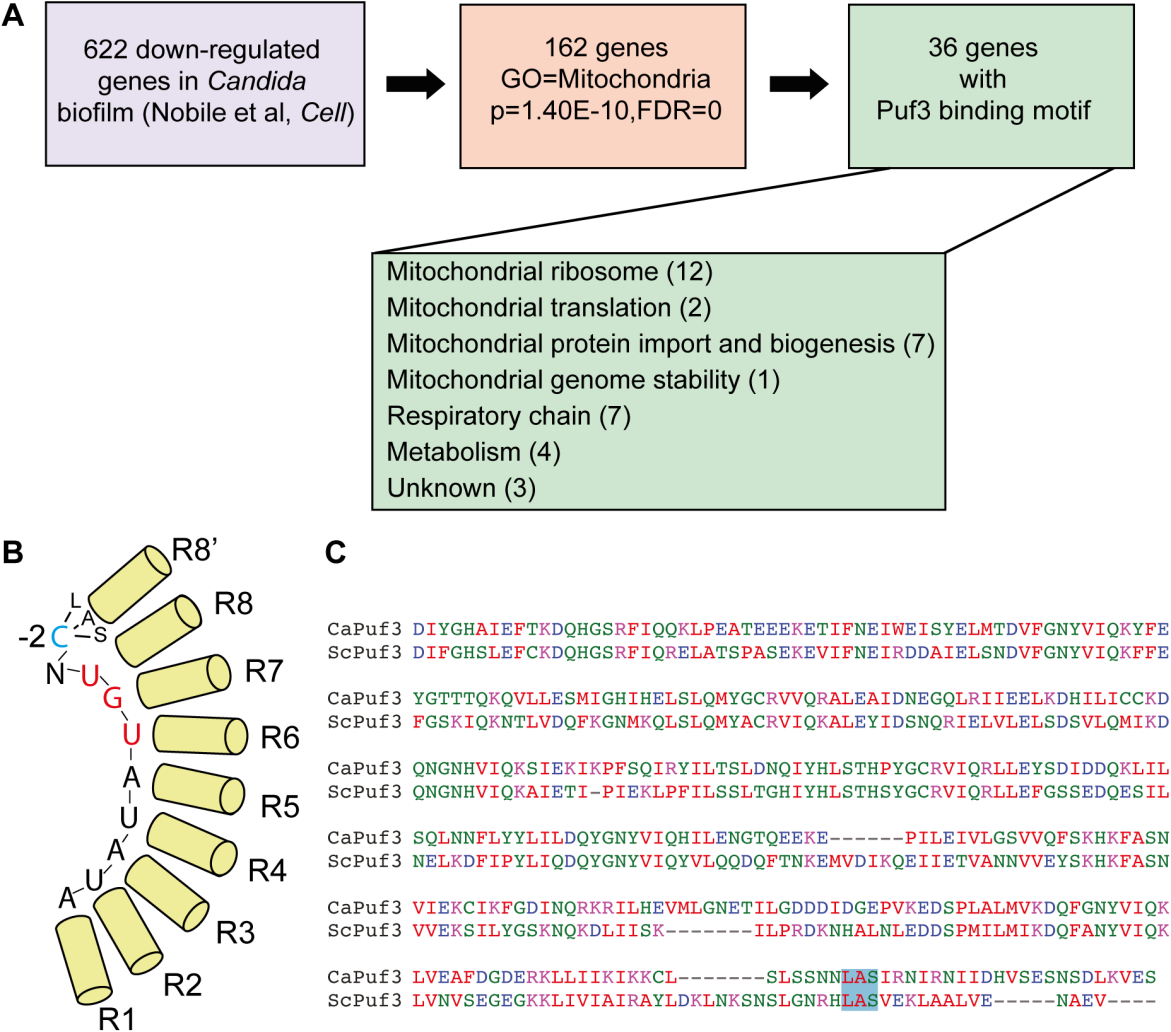
Biofilm mRNA targets of the Pumilio RNA binding protein Puf3. (A) Analysis of the gene expression data from [14] revealed that 162 genes down-regulated in biofilms are annotated to GO “Mitochondrion”, and 36/162 contain a Puf3 recognition element in their 3’ UTR. The putative biofilm-regulated Puf3 targets include several important mitochondrial biogenesis factors, such as mitochondrial ribosomal subunits, proteins required for respiratory chain function and assembly, and proteins that belong to the mitochondrial protein import machinery. Gene Ontology analysis was performed using the tools in the Candida Genome Database. (B) Cartoon of the Puf3 RNA binding domain from *S*. *cerevisiae* showing binding to the core recognition element and the interaction with the -2 cytosine (based on the structure reported in [39]. (C) Alignment of the PUM domains of the *C*. *albicans* and *S*. *cerevisiae* Puf3 proteins containing the 8–8’ repeat. The LAS motif of the Puf3 binding pocket that interacts with the -2 cytosine is boxed in blue, and this binding pocket is conserved in the *C*. *albicans* protein (see also [66]).

Inspection of the Nobile *et al* data [14] revealed that of the 622 genes down-regulated in biofilms relative to growth in suspension, 162 are annotated to the GO term “Mitochondrion” (p = 1.40E-10, FDR≈0) (S1 Dataset and Fig 1A). Of these, based on inferred functions from homology to the model yeast *S*. *cerevisiae*, we judged that 29 genes are likely to have dominant functions in other organelles (S1 Dataset). For example, some of the genes encode glycolytic enzymes, enzymes of ergosterol biosynthesis in the endoplasmic reticulum, cytoplasmic ribosome subunits and translation factors, and proteins with nuclear roles (S1 Dataset). In several cases, their annotation to mitochondria is based on proteomics studies that found the proteins in the mitochondrial proteome [32,33]. However, caution has to be applied, particularly when abundant proteins, as well as structures associated with mitochondria, such as the endoplasmic reticulum and mitochondria-associated translation, are considered. Whether these proteins play a role in mitochondrial activity/biogenesis in *C*. *albicans* remains to be studied.

The remaining 133 genes were sorted based on their putative roles into two groups: “activity” (genes necessary for mitochondrial functions, such as metabolic enzymes) (68 genes) and “biogenesis” (genes necessary for building the organelle) (58 genes) (S1 Dataset; of note, 7 genes had unclear functions and were not assigned to either category). 36 of the 133 mitochondria-related genes, and about half of the “biogenesis” group (26/58) possess in their 3′ untranslated region (3′ UTR) a predicted binding site for the RNA binding protein Puf3 as defined in *S*. *cerevisiae*: (C/U/A)(A/G/C/U)UGUA(A/C/U)AUA (Fig 1A and S1 Dataset). Puf3 is a Pumilio family RNA binding protein which in *S*. *cerevisiae* controls mitochondrial biogenesis by impacting on the decay and subcellular localization to mitochondria of a network of mRNAs encoding mitochondrial proteins [34–37], reviewed in [38]. Our analysis therefore suggests for the first time that gene regulation in *C*. *albicans* biofilms, and more specifically mitochondrial biogenesis, might be regulated by posttranscriptional mechanisms.

The structure of the *S*. *cerevisiae* Puf3 RNA binding domain in complex with its cognate site from the *COX17* 3′ UTR has been solved [39]. Much like other PUF proteins, Puf3 uses the repeats that form the concave surface of its arc-shaped RNA binding domain to interact with the eight bases of the core RNA recognition motif. It discriminates its own targets from those bound by other yeast PUF family members by virtue of a binding pocket in repeat 8–8′ of the RNA binding domain that interacts with a 5′ cytosine at position -2 of the recognition element [39] (Fig 1B). Although a cytosine at -2 leads to high affinity binding [39], other nucleotides can be found at this position in Puf3 targets, as shown by the consensus sequence [34,35,40]. Primary sequence alignment showed conservation of the -2 cytosine-binding motif in repeat 8′ of the PUM domain of *C*. *albicans* Puf3 (encoded by C4_05370W or orf19.1795). (Fig 1C). This suggests that *C*. *albicans* Puf3 recognizes the same motif as its *S*. *cerevisiae* homolog.

**Fig 2:**
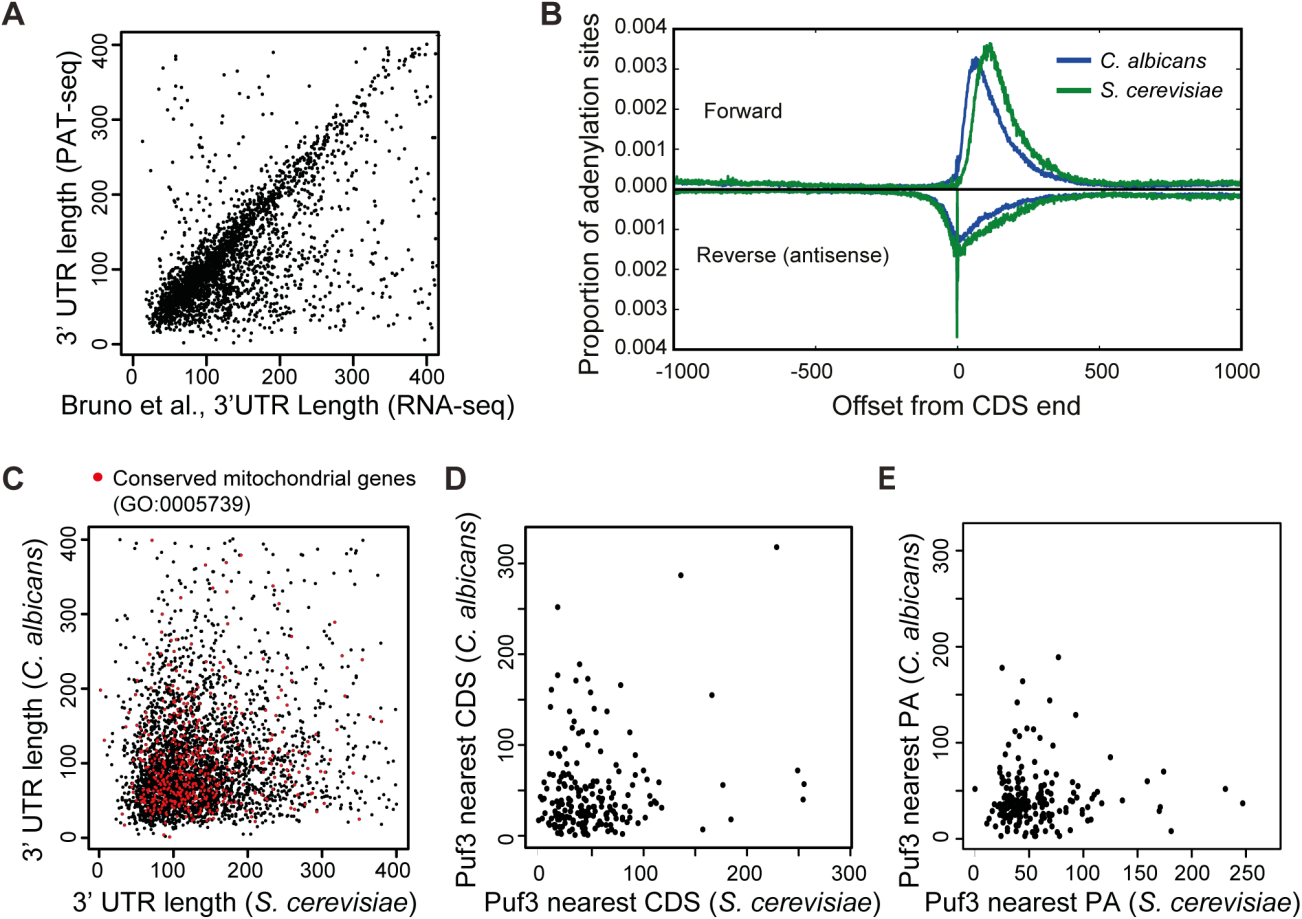
The 3′ UTR landscape of the *C*. *albicans* transcriptome. (A) Comparison of 3′ UTRs as determined by our study and Bruno *et al* [42]. Of the 3′ UTR that are called by both technologies, 84.5% are within 100 bases (r = 0.4684 (*p* much < 0.001, n = 2697. Of note, the correlation is highly significant because of the high numbers and would be classed as of moderate strength). Where there is a difference, it is due to filtering differences: we have used the most abundant 3′ UTR, whereas Bruno *et al* used the longest 3′ UTR for which there was evidence, including minor alternative 3′ UTR isoforms. (B) Graph showing 3′ UTRs are overall shorter in *C*. *albicans* than *S*. *cerevisiae*. The global positions of polyadenylation in the forward direction and, where it exists, the position of any anti-parallel overlapping adenylated RNA running in the reverse direction. Note any effect of filtering is avoided by this approach as all adenylation sites are utilized in this comparison (370997 and 201547 sites in *C*. *albicans* and *S*. *cerevisiae* respectively). (C) Comparison between the 3′ UTR lengths of the 3552 orthologous genes between *C*. *albicans* and *S*. *cerevisiae*. Genes annotated to GO “Mitochondrion” are labeled in red. (D) Comparison of the distance of the Puf3 binding site in putative mRNA targets conserved between *S*. *cerevisiae* and *C*. *albicans* relative to the closest coding sequence (CDS). (E) Comparison of the distance of the Puf3 binding site in putative mRNA targets conserved between *S*. *cerevisiae* and *C*. *albicans* relative to the polyadenylation site (PA).

To better define the Puf3-regulon in *C*. *albicans*, we performed a bioinformatics search for the (C/U/A)(A/G/C/U)UGUA(A/C/U)AUA recognition element in 3′ UTRs genome-wide. Firstly, we precisely defined the landscape of 3′ UTRs across the *C*. *albicans* transcriptome with a new 3′ sequencing technology that we developed called PAT-seq [41]. The 3' UTRs were called based on the most highly expressed peak within 400 bases of the end of the coding sequence, and not lying within a following gene on the same strand. Previous RNA-seq data has been informative in determining 3′ UTRs of *C*. *albicans* transcripts [42], and our mapping correlates well with the study of Bruno *et al* (Fig 2A). The apparent extension in length of 3′ UTRs in the Bruno *et al* data [42] is due to alternative adenylation that is present at lower abundance than the major peak called by our approach. The distinction between alternative 3′ UTR length isoforms is not easily extracted from regular RNA-seq, but is sensitively detected by PAT-seq. Moreover, with our technology we mapped 4862 3′ UTRs (or 78% of the transcriptome), and could map an additional 2006 3′ UTRs, beyond the annotations published by Bruno *et al* (S2 Dataset). Files to display 3′ UTR positions (including alternate isoforms where they exist) in CGD gbrowse are available (see Materials and Methods). We performed an equivalent analysis in *S*. *cerevisiae*, where we could map 5402 3′ UTRs. Comparison of positions of adenylation with *S*. *cerevisiae* showed that in *C*. *albicans* 3′ UTRs are overall shorter. The offset between convergent and overlapping 3′ UTRs is also slightly shorter, with both of these features reflecting a higher level of compaction of the *C*. *albicans* genome (Fig 2B). Despite this global similarity between 3′ UTR length distributions, the absolute 3′ UTR length in orthologous genes is not conserved between the two species (Fig 2C). Analysis of gene ontology enrichment (GO Function) in the *C*. *albicans* dataset, in windows of 50 bases of 3′ UTR length and at *p* < 0.0001, identified enrichment of very broad GO terms, such as “binding”, “anion binding”, “ATP binding”, “transferase activity”, “pyrophosphatase activity”, with one exception being “structural constituent of the ribosome” in the 50–99 bases 3′ UTR length group (S3 Dataset). An equivalent analysis of the *S*. *cerevisiae* dataset similarly showed broad terms, with the exception being “electron carrier activity” that mapped to 3′ UTRs longer than 200 bases (S3 Dataset). To further address if there is conservation of 3′ UTR lengths between the two yeasts related to gene function, we mapped genes with mitochondrial functions in Fig 2C (shown as red dots). However, no correlation was seen in regards to 3′ UTR lengths for mitochondria-related transcript between the two yeast species.

**Fig 3:**
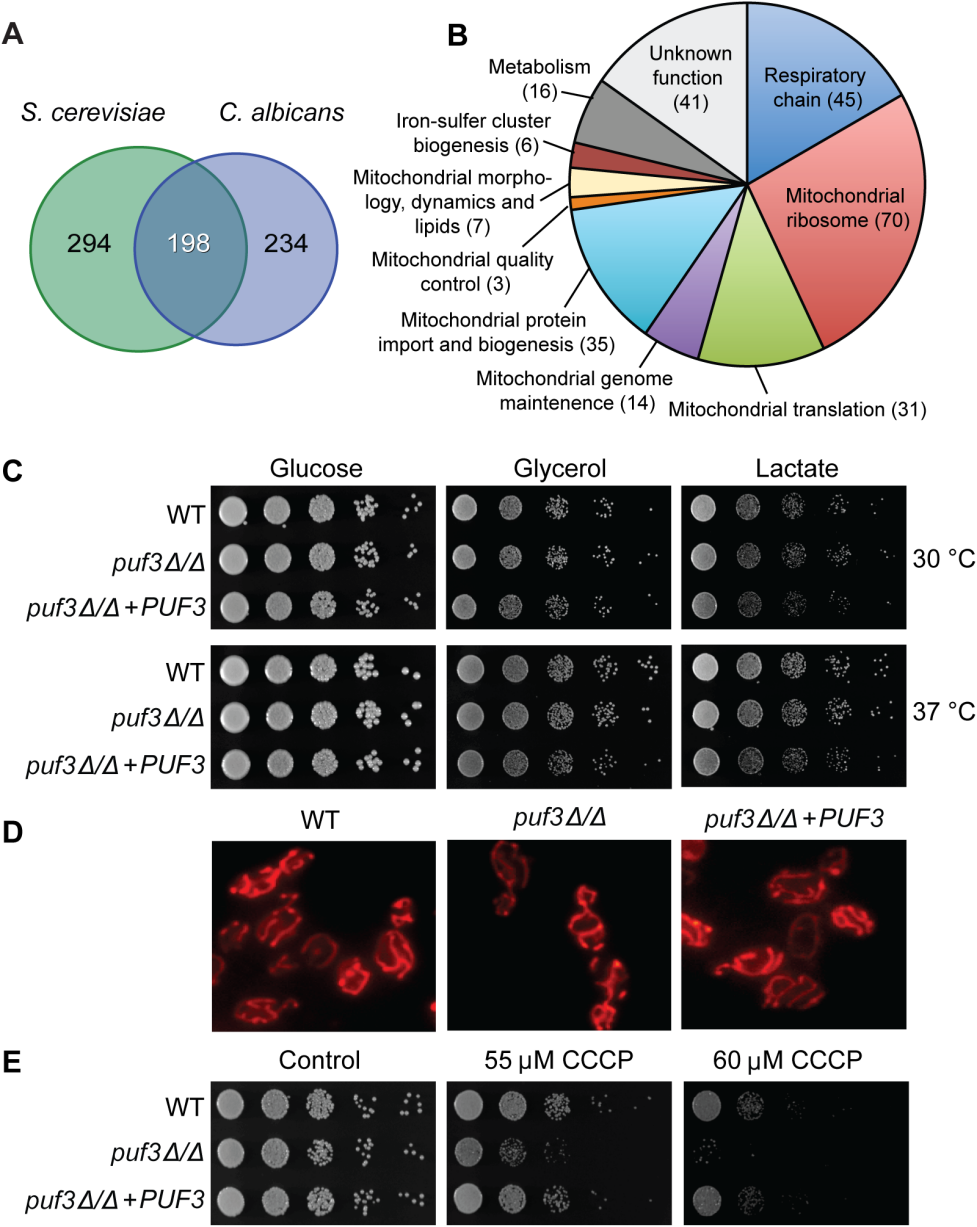
The *C*. *albicans* Puf3 regulon and mitochondrial roles. (A) Venn diagram showing the number of mRNAs with a Puf3 binding motif in the 3′ UTRs in *S*. *cerevisiae* and *C*. *albicans* (only genes which contain orthologs in both species are depicted here). (B) Functional groupings of the Puf3 regulon in *C*. *albicans*. The data used to produce this chart is shown in S4 Dataset. (C) Growth of *C*. *albicans* wild type and *puf3Δ/Δ* mutant on plates supplied with glucose, glycerol or lactate. Ten-fold serial dilutions were made starting from OD_600_ = 0.5, and plates were photographed after 2 days of growth. (D) Mitochondria in the indicated strains were stained with MitoTracker and imaged as described in Materials and Methods. (E) *C*. *albicans* growth on plates was tested as in (C), in the presence or absence of CCCP. Growth was observed on glycerol plates where mitochondrial function is essential.

Armed with the 3′ UTR landscape for the *C*. *albicans* transcriptome, we identified a total of 555 putative Puf3 targets (S2 Dataset). For comparative purposes, we searched the *S*. *cerevisiae* genome in an equivalent manner and identified 671 genes with Puf3 binding sites in their 3′ UTR (S2 Dataset). In 3′ UTRs, the Puf3 motif occurs two or three times as often as random motifs of the same composition. However in the genomes as a whole Puf3-binding sequences are not more prevalent than random motifs of the same composition (2751 instances in *S*. *cerevisiae*, and 3463 instances in *C*. *albicans*). Of the 555 putative Puf3 targets in *C*. *albicans*, 432 (77.8%) have an ortholog in *S*. *cerevisiae* (S2 Dataset, Fig 3A). The number of genes where both species have the Puf3 motif is 198 (Fig 3A). This correlation is highly significant by Fisher’s Exact Test, as similar analysis with a random shuffling of the motif generally produces a value less than 3 in common. Therefore, the Puf3 motif is much more highly conserved than shuffled motifs. The 3′ UTR length of the shared mRNAs with the Puf3 motif is not conserved (S1 Fig, red dots). Furthermore, the position of the Puf3 binding site is not preserved relative to the stop codon or the polyadenylation site between the two species (Fig 2D and 2E).

Within the predicted Puf3 targets in *C*. *albicans* 268 genes or 48.3% are assigned to the GO term “Mitochondrion” (p = 6.12e-141, FDR≈0) (S4 Dataset). With the same logic as described in the analysis of the biofilm-related genes, we judged that 12 of these genes encode proteins with predominant roles in another subcellular location. These are indicated in S4 Dataset, but for ease of comparison of the conserved and divergent putative Puf3 targets between *C*. *albicans* and *S*. *cerevisiae*, all analyses were performed with the 268-gene set. Inspection of the 198 conserved putative Puf3 targets between these two yeasts revealed that 177 belong to GO Mitochondrion (p = 126e-121, FDR≈0, S2 Dataset). In other words, the vast majority of the 198 conserved putative Puf3 targets belong to the mitochondrial network, and there is little conservation outside of that (Fig 3A). Cytosine at -2 is dominantly found in both *C*. *albicans* (115/198) and *S*. *cerevisiae* (127/198), however less than half (84/198 mRNAs or ≈ 42.4%) have a -2 C in both species (S2 Dataset). The vast majority of genes in the *C*. *albicans* Puf3-dependent mitochondrial network encode functions required for organelle biogenesis, rather than metabolic functions, including almost the entire set of proteins that constitute the mitochondrial ribosome (Fig 3B and S4 Dataset). Our results are consistent with a previous bioinformatic analysis that led to the proposition that Puf3 is an important regulator of mitochondrial biogenesis in the *Saccharomycotina* group of fungi [43].

### In *C*. *albicans*, Puf3 regulates mitochondrial biogenesis during growth in both glucose and the physiologically relevant carbon source lactate

Unlike the *S*. *cerevisiae puf3Δ* mutant, which shows reduced growth on non-fermentable carbon sources [34,43], the *C*. *albicans* homozygous deletion mutant *puf3Δ/Δ* was able to grow as well as the wild type strain in all carbon sources tested: glucose, glycerol and lactate (Fig 3C). Moreover, the mutant did not display any observable changes in mitochondrial morphology (Fig 3D). However, a mitochondrial role for Puf3 in *C*. *albicans* was revealed under mitochondrial stress. The mutant was hypersensitive to carbonyl cyanide *m*-chlorophenylhydrazone (CCCP), which uncouples electron transport through the respiratory chain from ATP synthesis (Fig 3E). This phenotype was complemented by re-introduction of the wild type *PUF3* gene into the mutant genome (Fig 3E).

**Fig 4:**
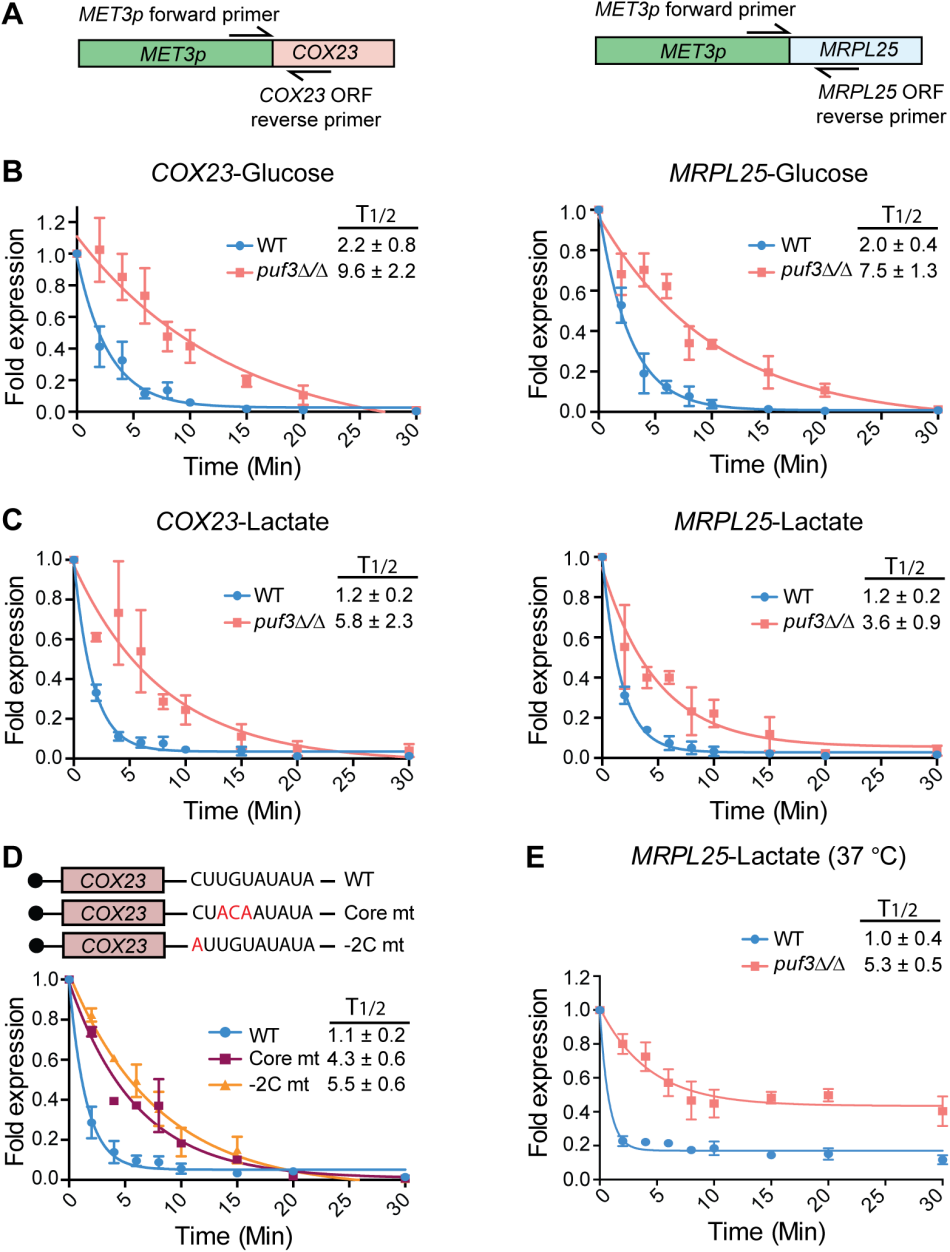
*C*. *albicans* Puf3 is a repressor of mRNA stability in glucose and lactate growth conditions. **(**A) A cartoon depicting the location of primers used for specific amplification of the *MET3p*-driven *COX23* and *MRPL25* genes. Detection of this allele was specific, as demonstrated in S3 Fig. (B) qPCR showing time dependent decay of *COX23* and *MRPL25* genes following transcriptional shutdown of *MET3p* in glucose. Fold expression was represented as ratio of expression levels for each time point after dividing with the expression levels at time 0. Decay curves were obtained with the nonlinear regression (curve fit) method using the exponential, one phase decay equation in the GraphPad “Prism 6” software. The half-life (T1/2) was also calculated using this equation by plotting the decay curve of 3–4 biological replicates separately, and is shown as the average ± standard error. (C) The experiments were performed as described in (B), but with lactate as the carbon source. Data represent the average and standard error from 4 biological replicates. (D) The cartoon depicts mutations in the core and -2C positions of the Puf3 binding motif in the *COX23* 3′ UTR. Decay curves are from wild type *C*. *albicans* strains expressing either a *COX23* construct with either the wild type or the mutant Puf3 recognition element. The experiments were performed as in (B). Strains were grown in lactate media and shown are results of 2 biological replicates. (E) The experiment was performed as in (C), but concomitant with addition of methionine and cysteine to inhibit transcription, the temperature was raised to 37°C. Shown are results of 3 biological repeats.

PUF proteins negatively impact on mRNA stability by recruiting deadenylases such as Ccr4, which digest the poly(A) tail and initiate decay, reviewed in [38]. Therefore, we next tested mRNA half-lives in the absence of Puf3 in *C*. *albicans*. Attempts to use the transcriptional inhibitor 1,10 phenantroline in *C*. *albicans* failed (no repression of gene transcription was observed for a prolonged time, S2A Fig). Another transcriptional inhibitor, thiolutin, was effective in inhibiting transcription only at very high doses, and had a stabilizing effect on mRNA (S2B and S2C Fig). To circumvent these technical problems with transcriptional inhibitors, we placed two Puf3 targets, *MRPL25* encoding a mitochondrial ribosomal subunit, and *COX23* encoding a cytochrome c oxidase assembly factor, under the repressible *MET3* promoter. In this set up, transcription is “on” in the absence of methionine and cysteine in the medium, whereas addition of methionine and cysteine results in rapid repression. In these strains, only one copy of the Puf3-dependent gene is placed under the *MET3* promoter, and therefore primers were used to specifically detect this copy and not the endogenous gene (Fig 4A). This strategy was chosen over deleting the second copy because of the high impact of mitochondrial mutations on *C*. *albicans* fitness due to its petite negative nature [44]. Control experiments showed that the detection of the *MET3p*-controlled transcripts of *MRPL25* and *COX23* by quantitative PCR experiments was highly specific (S3 Fig), and these strains had no observable growth defects in any of the carbon sources tested (S4 Fig).

To address how Puf3 impacts on the stability of its putative mRNA targets, the experiment was performed as follows: the gene was repressed initially, then transcription was induced for 10 minutes, followed by repression and monitoring of the decay of the newly synthesized mRNA (see Materials and Methods). The experiments were done in glucose, and in the non-fermentable carbon source lactate, which is physiologically important for *C*. *albicans* in human host niches [7]. The half-lives of these transcripts in wild type cells were not affected by carbon source, as in both glucose and lactate the mRNAs were rapidly decayed with similar kinetics (Fig 4B and 4C). During growth in either glucose or lactate, deletion of *PUF3* resulted in slower decay of the *MRPL25* and *COX23* transcripts (Fig 4B and 4C). We demonstrate that the effect of *PUF3* is direct by showing that mutations of the Puf3 binding site in the 3′ UTR of *COX23* (in the core element or a -2 cytosine to alanine mutation), phenocopied the deletion of *PUF3* in otherwise wild type strains (Fig 4D). The result with the -2C to A mutation is consistent with an important function of -2C for Puf3 binding in *C*. *albicans*.

**Fig 5:**
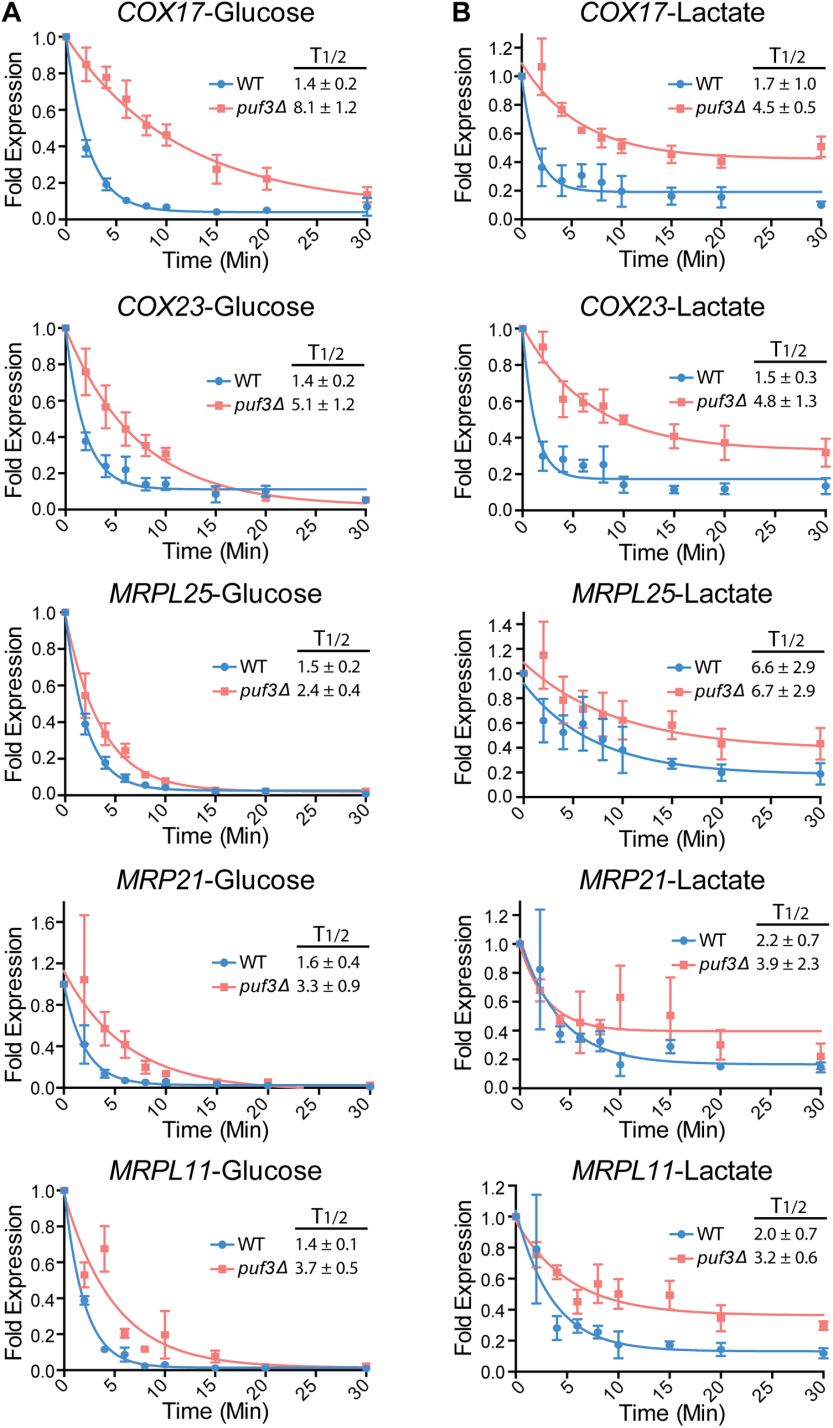
Posttranscriptional regulation of the mitochondrial ribosome and the *COX* genes by carbon source and Puf3 in *S*. *cerevisiae*. Decay of the indicated mRNAs was measured in wild type and *puf3Δ* strains grown in glucose (A) or lactate (B) following transcriptional repression at 37°C. The decay curves and half-life (T1/2) were calculated as in Fig 4. The data are shown as the average and standard error of 2–4 biological replicates.

Our results were somewhat surprising in light of previous publications in *S*. *cerevisiae* that showed that: a) transcripts encoding mitochondrial proteins are stabilized during growth of a wild type strain in non-fermentable carbon sources compared to growth in glucose, and b) Puf3 represses mRNA stability only in glucose, but not in non-fermentable carbon sources [36,45]. While previous studies in *S*. *cerevisiae* used several non-fermentable carbon sources, lactate was not directly tested. To test the effects of lactate in *S*. *cerevisiae*, we made use of a strain that carries an RNA polymerase II temperature sensitive mutation (*rpb1-1*) which allows for repression of transcription at 37°C (wild type and *puf3* mutant, described in [36]). Similarly to *C*. *albicans*, in *S*. *cerevisiae* deletion of *PUF3* had a stabilizing effect in both glucose and lactate on two transcripts that encode cytochrome c oxidase assembly factors: *COX17* and *COX23* (Fig 5). Moreover, in wild type cells the half-life was similar for these two transcripts in glucose and lactate media (compare Fig 5A and 5B). The situation with the mRNA encoding the mitochondrial ribosomal subunit *MRPL25* was different. Firstly, unlike in *C*. *albicans*, in *S*. *cerevisiae* the *MRPL25* transcript was stabilized in lactate compared to glucose in wild type cells (compare Fig 5A and 5B). Secondly, while deletion of *PUF3* had a stabilizing effect in glucose (albeit less pronounced than what is seen in *C*. *albicans*), in lactate the half-life for *MRPL25* was the same in wild type and *puf3Δ* mutant cells (Fig 5A and 5B). For two other transcripts encoding mitochondrial ribosomal proteins, *MRP21* and *MRPL11*, in wild type cells mRNAs decay was also fast in glucose media and slower in lactate, although stabilization in lactate was less pronounced than what was observed for *MRPL25* (Fig 5A and 5B, bottom two graphs). Deletion of *PUF3* resulted in stabilization of *MRP21* and *MRPL11* in glucose (Fig 5A), and also some stabilization, particularly for *MRPL11*, was observable in lactate (Fig 5B). As controls, we assayed three transcripts with mitochondrial functions, which do not contain Puf3 binding sites in the 3′ UTR: *MMF1*, *FUM1* and *OAC1*. Deletion of *PUF3* had no effect on the decay of these three transcripts in either glucose or lactate (S5 Fig). Therefore, mRNAs that do not contain a Puf3 binding motif do not respond to deletion of *PUF3*, suggesting that the observed stabilization of the *COX* and *MRP* genes in the *puf3Δ* mutant is specific.

In *S*. *cerevisiae* cells grown in lactate the decay of all tested transcripts (Puf3-dependent and Puf3-independent) was altered compared to glucose. Moreover, it appeared that, after initial decay, a subpopulation of the transcripts remained stable over the course of the experiment (see Fig 5). This was not observed in *C*. *albicans* (Fig 4). Unlike in *C*. *albicans*, in *S*. *cerevisiae* transcription was inhibited by a shift to 37°C, and we wondered whether the combination of lactate media plus the temperature shift might be contributing to the nature of the decay. To test this, we repeated the experiment with the *MET3p-MRPL25* strains of *C*. *albicans* in lactate, but shifting the strains to 37°C concomitant with addition of methionine and cysteine to the media to repress transcription. Indeed, in these conditions in *C*. *albicans* also we observed fast initial decay and a subpopulation of transcripts that remained stable over the course of the experiment (Fig 4E). This result indicates that the combination of lactate and temperature shift results in altered mRNA decay for a subpopulation of transcripts. Importantly, consistent with what was observed when the experiment was done at room temperature, and in contrast to the result from *S*. *cerevisiae*, in *C*. *albicans* the half life of *MRPL25* in the wild type remained very short in lactate at 37°C, and clear stabilization was seen in the *puf3Δ/Δ* mutant (Fig 4E).

A possible explanation for the altered decay curves observed in lactate at 37°C is the presence of two sub-populations of mRNAs: one that is decayed faster and one that is decayed slower through the use of alternative polyadenylation sites. This would be carbon-source dependent, as it is only seen in lactate and not in glucose (Fig 5). To test this, we used extension poly-A test (ePAT) [46] on three transcripts in *S*. *cerevisiae*, *COX17*, and two other mRNAs that encode mitochondrial proteins: *TOM70*, which contains a Puf3 binding site in its 3′ UTR and *OM14*, which does not. No alternative 3′ UTRs were observed for *COX17* in glucose or lactate media, arguing against the use of alternative polyadenylation sites in this transcript dependent on carbon source (S6 Fig). However, both *TOM70* and *OM14* revealed a transcript with a shorter 3′ UTR that was stabilized in lactate media (S6 Fig). Collectively, these results show that the use of polyadenylation sites can be influenced by carbon source, but not necessarily in relation to Puf3 function.

### Posttranscriptional gene regulation impacts on the development of *C*. *albicans* biofilms

**Fig 6:**
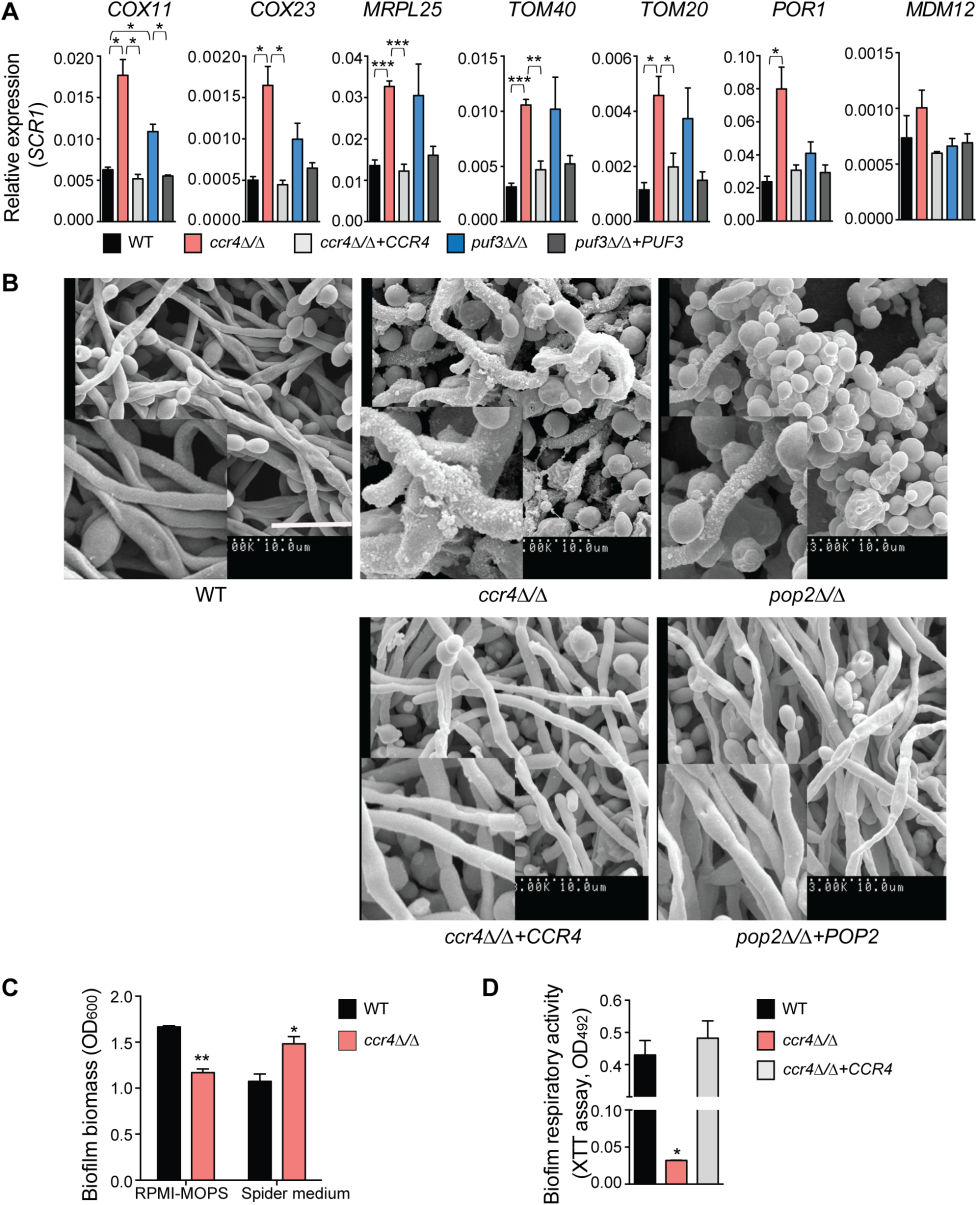
The mRNA deadenylase Ccr4 regulates extracellular matrix production in biofilms. (A) qPCR analysis of the expression of mitochondrial biogenesis genes in biofilms grown for 48 h in Spider medium. *SCR1* RNA was used for normalization. Error bars are ± standard errors of the average of 3 biological replicates. *P* values are as follows: *** <0.001, ** <0.01, * <0.05. Additional genes are shown in S7 Fig. (B) Scanning electron microscopy of biofilms formed by *C*. *albicans* wild type (DAY185), *ccr4Δ/Δ* and *pop2Δ/Δ* mutants and their respective complemented strains. Mature biofilms (48 h) grown in Spider medium were assessed. Experiments were repeated at least twice and inset boxes are regional amplifications. Scale bar = 10 μm. Similar results were obtained when biofilms were grown in RPMI-MOPS or YNB (S9 Fig). (C) The data for RPMI-MOPS are from the control samples in the experiments performed to assess biofilm susceptibility to zymolyase (Fig 7B). Biofilms were grown for 48 h in the indicated media. Total biofilm biomass was determined by staining with crystal violet. Bars represent averages ± standard errors from three biological replicates. *P* value of the difference between WT versus *ccr4Δ/Δ* is shown as ** <0.01 in RPMI-MOPS and *<0.05 in Spider media. (D) Metabolic activity of *C*. *albicans* biofilms was determined using the XTT reduction assay. Results were calculated from three independently grown biofilms for each of the strains assayed in technical duplicates. The error bar represents standard errors. *P* value of the difference between WT and *ccr4Δ/Δ* is shown as * <0.05.

As shown in Fig 1, a proportion of the genes that contain a Puf3 binding site is differentially expressed in *C*. *albicans* biofilms. Therefore, we next investigated the roles of posttranscriptional gene regulation in biofilm formation. In addition to Puf3 (this study), we have previously shown that the Ccr4 mRNA deadenylase is involved in mitochondrial function in *C*. *albicans* [29]. Puf3 and Ccr4 are functionally linked. Puf3, like other PUF proteins, can bind to the Ccr4-NOT complex [47], which is thought to be the mechanism by which Ccr4 is recruited to PUF-dependent mRNAs for poly(A) tail deadenylation and decay, reviewed in [38]. Consistent with a role for these posttranscriptional regulators in biofilm gene expression, deletion of either *PUF3* or *CCR4* resulted in differential expression of mitochondria-related genes in *C*. *albicans* biofilms (Fig 6A). *CCR4* had a more pronounced effect than *PUF3*, as would be expected for a major mRNA deadenylase that is recruited to transcripts by multiple RNA binding proteins. Control genes that do not contain a Puf3 binding site in the 3′ UTR (*POR1*, *MDM12*, *MDM10*) were not up-regulated in the *puf3Δ/Δ* mutant (Fig 6A and S7 Fig), although *POR1* was significantly up-regulated in the *ccr4Δ/Δ* mutant in line with a broader role for Ccr4 in gene expression (Fig 6A and S7 Fig). The levels of *HWP1* and *TEF1*, which are not related to mitochondria, were not up-regulated in either mutant (S7 Fig), arguing against non-specific higher levels of mRNAs due to lack of a major mRNA decay pathway.

**Fig 7:**
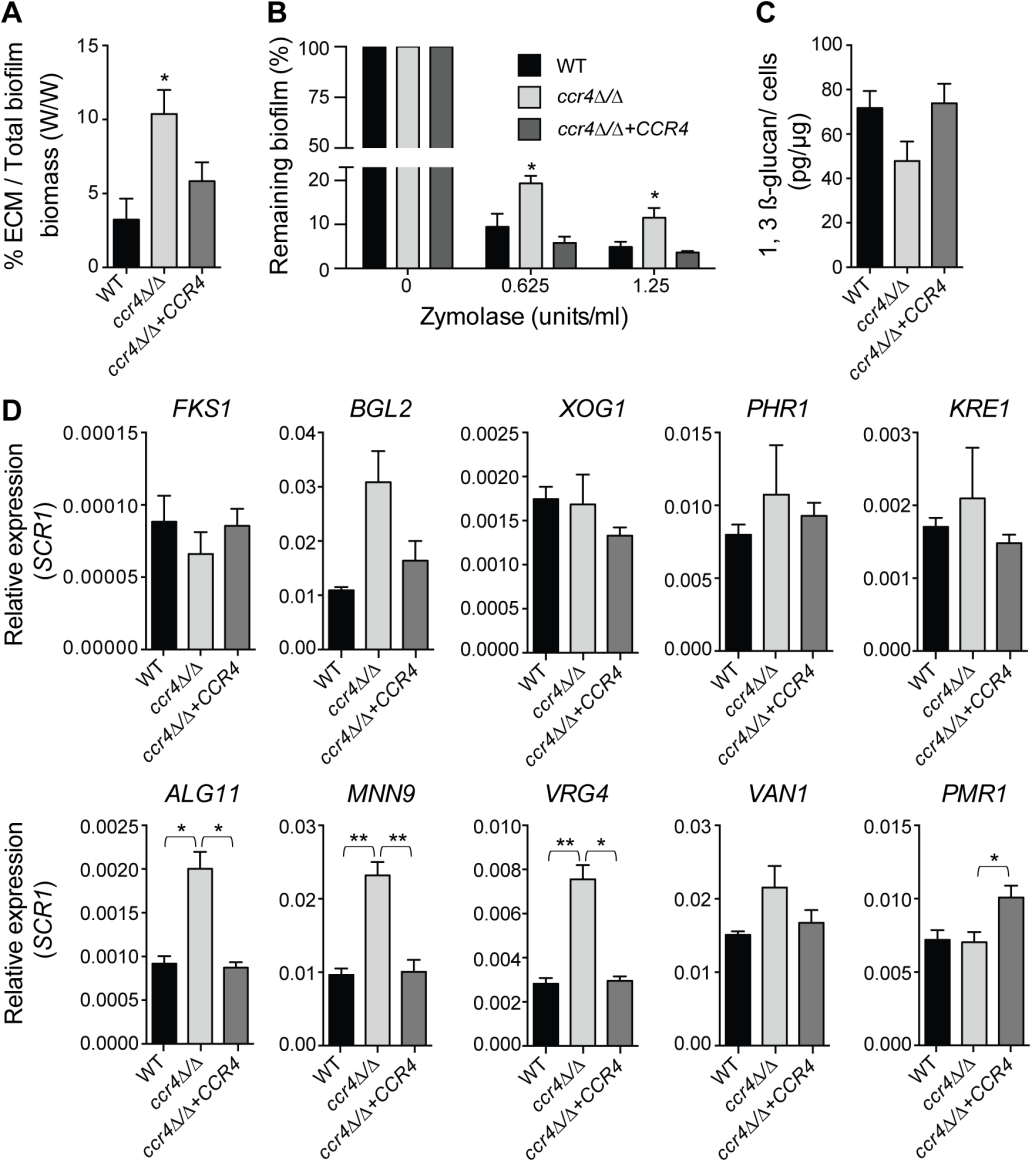
Effects of *CCR4* on the extracellular matrix and biofilm stability. (A) Quantification of total extracellular matrix (ECM) in *C*. *albicans* biofilms. Biofilms were grown in Spider medium and collected after 48 h. The proportion of ECM was calculated relative to the total biofilm biomass (cells + ECM), as determined by dry weight measurement (see Materials and Methods). Results were calculated from three biological repeats in technical triplicates. Shown are the averages and the standard errors. *P* value of the difference between WT and *ccr4Δ/Δ* is shown as * <0.05. (B) Biofilms were grown in 96-well microtiter plates in RPMI-MOPS for 24 h and then exposed to zymolyase (20T) prepared in RPMI-MOPS + 0.9% NaCl (1:1 ratio) for 24 h. The remaining biomass was quantified by crystal violet staining. Results were calculated from three biological repeats in technical duplicates. Shown are the averages and the standard errors. *P* value of the difference between WT and *ccr4Δ/Δ* is shown as * < 0.05. (C) Matrix 1,3 β-glucan was quantified from biofilms grown in Spider medium as described in Materials and Methods. The yield of 1,3 β-glucan was calculated as the weight of 1,3 β-glucan (pg) per 1 μg of biofilm cells. Results were calculated from 6 biological repeats. Shown are the averages and the standard errors. Differences: WT versus *ccr4Δ/Δ*, *p* = 0.067; *ccr4Δ/Δ* + *CCR4* versus *ccr4Δ/Δ*, *p* = 0.062. (D) qPCR analysis for the indicated transcripts was performed on biofilms grown for 48 h in Spider medium and data normalized to *SCR1* RNA. Error bars are ± standard errors of the average of 3 biological replicates. *P* values are as follows: ** <0.01 or * <0.05.

Consistent with milder effects on gene regulation, and suggestive of compensatory effects, the *puf3Δ/Δ* mutant formed biofilms of wild type structure (S8 Fig). However, analysis of the *ccr4Δ/Δ* mutant revealed a clear role of posttranscriptional gene regulation in *C*. *albicans* biofilms. The biofilms formed by *ccr4Δ/Δ* showed altered structure in scanning electron micrographs, with an increase in yeast cells over filamentous cells and hyper-production of biofilm extracellular matrix material (Fig 6B). The effect on hyper-production of extracellular matrix was observed in several biofilm growth media (RPMI, Spider and YNB) (Fig 6B and S9 Fig), regardless of whether the mutant biofilm showed somewhat increased or decreased biomass (in Spider and RPMI respectively, Fig 6C). Therefore, the changes to biofilm extracellular matrix of the *ccr4Δ/Δ* mutant are unlikely to be caused by changes in growth rates and final biomass. Consistent with mitochondrial dysfunction, *ccr4Δ/Δ* mutant biofilms returned very low levels of respiratory activity using the XTT-reduction assay, which depends on mitochondrial respiration (Fig 6D). Similar biofilm phenotypes were displayed by *pop2Δ/Δ*, which is inactivated in the *POP2* subunit of Ccr4-NOT that is also essential for mRNA deadenylase activity (Fig 6B and S9 Fig). Quantification of extracted extracellular matrix relative to total biofilm biomass showed a ~2 fold increase in *ccr4Δ/Δ* mutant compared to wild type (Fig 7A), and the *ccr4Δ/Δ* biofilm was less sensitive to treatment with zymolyase preparations (Fig 7B). Zymolyase preparations contain 1,3 ß-glucanase, mannanase, protease and endochitinase activities [48] and are therefore likely to act on several components of the extracellular matrix [49]. Enzymatic activities that impact on glucan synthesis and remodeling have a major impact on biofilm matrix levels [15,16], and our previous work showed that in the absence of *CCR4* the relative levels of glucan in the cell wall are reduced [29]. Consistent with this, the level of matrix 1,3 ß-glucan as determined by the Glucantell kit was not higher in *ccr4Δ/Δ* biofilms (Fig 7C). Furthermore, genes encoding enzymes required for glucan synthesis and remodeling, including *BGL2*, *XOG1* and *PHR1* that have roles in 1,3 ß-glucan accumulation in the biofilm matrix [15], were not significantly up-regulated in *ccr4Δ/Δ* mutant biofilms (Fig 7D; we note that a trend toward higher levels of *BGL2* was observed). We further assayed five genes that regulate the level of mannan in the biofilm extracellular matrix [16]. Of these, three (*ALG11*, *MNN9* and *VRG4*) displayed significantly higher expression levels in *ccr4Δ/Δ* biofilms (Fig 7D), suggesting that the activity of Ccr4 impacts on the expression of enzymatic activities that control biofilm matrix production.

### Mitochondrial activity in the development of *C*. *albicans* biofilms

**Fig 8:**
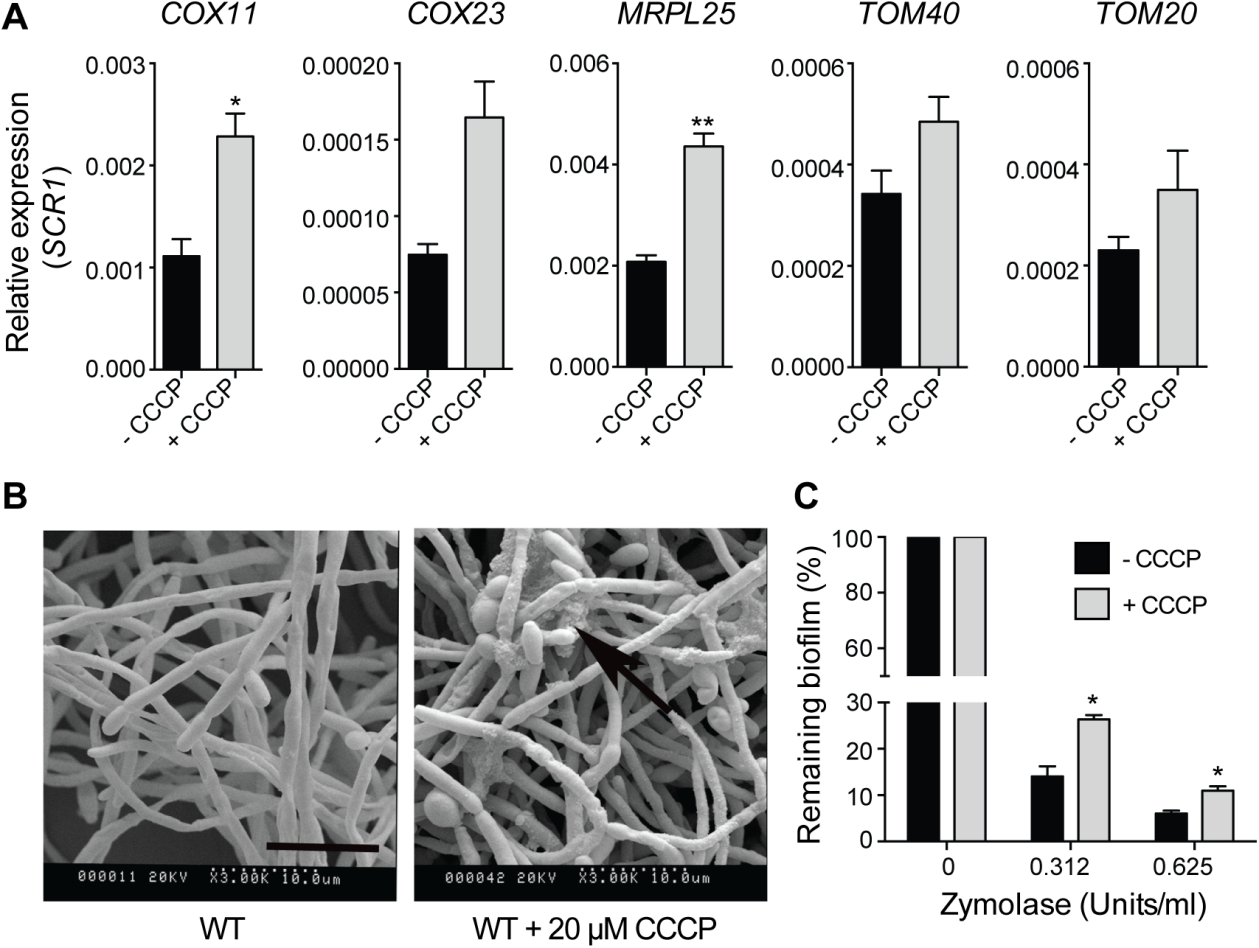
Uncoupling of mitochondrial oxidative phosphorylation stimulates biofilm matrix production. The wild type stain DAY185 was used for these experiments. (A) qPCR analysis of the expression of the indicated mitochondrial biogenesis genes in 48 h grown biofilm samples upon treatment with CCCP. Error bars are ± standard errors of the average of three biological replicates. *P* values are as follows: ** <0.01, * < 0.05. (B) SEM of biofilms in the presence or absence of 20 μM CCCP. The ECM is indicated with black arrows. Scale bar = 10 μm. (C) The susceptibility to zymolyase of CCCP-treated biofilms grown in RPMI-MOPS was determined by crystal violet staining. Results were calculated from three biological repeats in technical duplicates. Shown are the averages and the standard errors. *P* value is * < 0.05.

One of the main stresses experienced by biofilm cells is hypoxia [50,51], and Ccr4 has been recently implicated in the response of *C*. *albicans* to hypoxia [52]. We therefore hypothesized that, additional to regulation of factors with roles in matrix carbohydrate accumulation, changes to mitochondrial activity in *ccr4Δ/Δ* biofilms, and potentially hypoxic adaptation might be responsible for the observed biofilm phenotypes of the mutant. Treatment with CCCP, which uncouples mitochondrial oxidative phosphorylation, mimics the early effects of hypoxia on the level of the genome-wide transcriptional response [52]. Therefore, we analyzed the structural features of biofilms in the presence of CCCP. For these experiments we predominantly used RPMI medium, because it supported biofilm growth significantly better than Spider medium upon mitochondrial inhibition. The dose of 20 μM CCCP is in line with previous studies in *S*. *cerevisiae* [53], and was effective in causing mitochondrial stress: similarly to *ccr4Δ/Δ* biofilms, treatment with CCCP led to up-regulation of mRNAs with mitochondrial functions (Fig 8A), which is a known consequence of mitochondrial perturbation [29,54]. CCCP had an effect on biofilm growth (S10 Fig), but nevertheless a complex biofilm that structurally resembled the wild type formed (Fig 8B). Similar to deletion of *CCR4*, treatment of biofilms with CCCP led to the accumulation of extracellular matrix (Fig 8B), and somewhat greater stability upon treatment with zymolyase (Fig 8C). These results support the proposition that mitochondrial dysfunction triggers ECM accumulation in biofilms.

## Discussion

Regulatory networks orchestrated by transcription factors have been widely studied in fungal species and metazoans, from the perspective of pathway regulation as well as evolutionary conservation and/or rewiring. An example relevant to our study is the report of Nobile *et al* of a highly interconnected network of transcription factors that regulate biofilm development in *C*. *albicans* [14]. We show here that, beyond transcriptional control, posttranscriptional gene regulation impacts on biofilm formation in *C*. *albicans*. Our data suggest that the mechanism involves regulation of mitochondrial function, as well as control over the expression of cell wall genes that are involved in biofilm matrix production. We provide the first functional data supporting regulatory rewiring of posttranscriptional mechanisms that mediate mRNA stability between *S*. *cerevisiae* and *C*. *albicans*. We further describe a novel function for the Ccr4 mRNA deadenylase and mitochondrial metabolism in a key biofilm protection mechanism: the accumulation of the extracellular matrix.

### Insight into the 3′ UTR landscape and posttranscriptional regulation in *C*. *albicans*

Following transcription, mRNAs can be organized into co-regulated networks termed “*posttranscriptional operons*”, through sharing of recognition elements bound by RNA binding proteins [55,56]. Compared to transcription, little is known about the function and evolution of posttranscriptional mRNA networks in either model or pathogenic fungi. We performed 3′-based RNA-seq that allowed us to map precisely 3′ UTRs on 4862 *C*. *albicans* transcripts, 1/3 of the transcriptome more than what has been previously defined [42]. We found no general conservation of 3′ UTR lengths between *S*. *cerevisiae* and *C*. *albicans* mRNAs. Also, while the regulation of the functional network of mitochondria-related mRNAs by Puf3 is conserved between *S*. *cerevisiae* and *C*. *albicans* (this study and [43]), we show that the location of the Puf3 binding site relative to the stop codon or polyadenylation site is not conserved, arguing that these features might not be critical for Puf3-dependent regulation. This could mean a difference with another related PUF protein, Puf5 in *S*. *cerevisiae*, for which the position of the binding motif relative to the termination codon impacts on repression [57]. As in *S*. *cerevisiae*, in *C*. *albicans* cytosine at position -2 in the recognition element is prevalent in the mitochondria-related putative Puf3 targets, and our data shows that -2C is important for repression of *COX23* (Fig 4E). However, our analysis shows that the -2C is conserved in only about 50% of the mRNAs that contain a Puf3 recognition motif in both yeasts, suggesting it might not be as critical for Puf3-dependent regulation.

Our data sheds light on the regulatory rewiring of gene expression in fungi. This has mostly been studied at the level of gene transcription, and information on the evolution of posttranscriptional mechanisms is scarce. Bioinformatic studies of the distribution of Puf3 and Puf4 binding motifs have suggested that their roles might have changed during fungal evolution [43,58]. However, biological insight into the regulation and relevance for organismal biology of these posttranscriptional networks in distinct species is lacking. A major functional group containing Puf3 binding sites is the mitochondrial ribosomal genes. In an elegant example of evolutionary change, genes encoding mitochondrial ribosomal proteins have been transcriptionally rewired between the Crabtree positive *S*. *cerevisiae* and the Crabtree negative *C*. *albicans* by loss of a promoter element in *S*. *cerevisiae* that enabled the uncoupling of the regulation of the cytoplasmic ribosome and rRNA biogenesis genes from that of the mitochondrial ribosome [59]. Our data shows differences in the regulation of mRNA stability of the mitochondrial ribosomal subunit *MRPL25* between *C*. *albicans* and *S*. *cerevisiae*, suggesting that mitochondrial ribosomal gene expression has been rewired at multiple levels of control during fungal evolution. In glucose, *MRPL25* was rapidly decayed in both yeasts. In lactate the decay of *MRPL25* remained rapid in *C*. *albicans*, but it was slower in *S*. *cerevisiae* (Figs 4 and 5). Moreover, in *C*. *albicans* deletion of *PUF3* caused stabilization of *MRPL25* in both glucose and lactate, whereas in *S*. *cerevisiae* this was seen only in glucose. We could show that these differences in posttranscriptional regulation of *MRPL25* by carbon source and Puf3 were not due to the use of a temperature shift to stop transcription in *S*. *cerevisiae*, as rapid decay and the effect of the *puf3* mutation were maintained in *C*. *albicans* when the experiment included a temperature shift. We further considered the possibility that transcriptional up-regulation of *MRPL25* in lactate media has caused a stoichiometry problem for the decay machinery in *S*. *cerevisiae*, thereby non-specifically slowing decay (of note, in *C*. *albicans* the gene is regulated by the *MET3* promoter and so it is dissociated from regulation by carbon source). However, we do not believe this to be the case: firstly, the induction of the *COX* genes and the *MRPL25* gene in lactate versus glucose was uniform for all three genes and *MRPL25* was induced the least (≈7 fold for *COX1*7, ≈5 fold for *COX23* and ≈4.3 fold for *MRPL25*); yet only for *MRPL25* we observed a change towards a longer half-life in lactate. Secondly, comparing transcript levels normalized to the loading control gene *SCR1* in *C*. *albicans* and in *S*. *cerevisiae* at the time of transcriptional repression (time point 0) showed similar relative levels in lactate media, and yet only in *S*. *cerevisiae* stabilization was observed. Different mitochondrial ribosomal subunit genes might be impacted to a different degree by carbon source and Puf3-dependent regulation: compared to *MRPL25*, a smaller effect of carbon source on the half life was observed for *MRP21* and *MRPL11*, and the transcripts were stabilized in the *puf3Δ* mutant in glucose, and a smaller effect was seen in lactate. Collectively our data suggest that the differences in mRNA stability control for the mitochondrial ribosomal subunits between *S*. *cerevisiae* and *C*. *albicans* reflect regulatory rewiring to enable fine-tuning of mitochondrial biogenesis with metabolic status and cellular energy requirements. Data with *MRPL25* suggest that this regulatory difference might be in part due to distinct control by carbon source over Puf3 activity in the two fungal species.

### Ccr4 and mitochondrial activity are new regulators of biofilm maturation and extracellular matrix production

A proportion of the mitochondria-related genes differentially expressed in *C*. *albicans* biofilms belongs to the Puf3-regulon (S1 Dataset and Fig 1A). A role for posttranscriptional gene regulation in biofilm maturation was revealed here by studying the consequences of inactivation of the main mRNA deadenylase *CCR4*, which mediates Puf3-dependent repression and has a dominant role in regulating mitochondrial activity in biofilms. While inactivation of both *CCR4* and *PUF3* results in higher levels of mitochondria-related genes, both of these factors have a positive role in mitochondrial biogenesis (this study and [29]). As we have proposed, this could reflect the roles of mRNA targeting to mitochondria for localized translation, and translational inhibition at the mitochondrial surface to assist co-translational protein import into mitochondria [38]. Drastic structural alterations were observed in *ccr4Δ/Δ* biofilms, with morphogenetic change towards more yeast-form cells (consistent with a role of Ccr4 in hyphal differentiation [29]), over-production of extracellular matrix and higher biofilm resistance to degrading enzymes. To our knowledge, Ccr4 is only the third gene expression regulator of biofilm matrix production in *C*. *albicans*, and the second negative regulator, the other one being the zinc-responsive transcription factor Zap1 [18]. Biofilms made by the *zap1* mutant resemble those made by the *ccr4* mutant in several aspects: they display hyper-production of extracellular matrix (although the precise components of the matrix regulated by these two factors differ), and there are more yeast-form cells compared to the hyphae-rich wild type biofilms [18]. Our study therefore adds a key posttranscriptional gene expression regulator as an important factor determining biofilm maturation.

To date, almost all of the genes known to affect *C*. *albicans* biofilm matrix relate functionally to cell wall integrity, reflecting the fact that cell wall carbohydrates β-glucans and mannans are important components of the matrix material [49]. In the case of Zap1, effects on the expression of glucan hydrolyases and metabolism/quorum sensing via expression of alcohol dehydrogenases have been suggested to play a role in matrix regulation [18]. In contrast to Zap1, Ccr4 does not regulate the levels of matrix 1,3 β-glucan, but instead several genes required for the accumulation of matrix mannan [16] were expressed at higher levels in biofilms of the *ccr4Δ/Δ* mutant. Given the prominent role of Ccr4 in both mitochondrial activity and cell wall integrity [29], and a link between mitochondrial function and the cell wall in *C*. *albicans* and other fungi [27], we further considered that modulation of mitochondrial function in biofilms due to hypoxia could be sensed as a stress signal, and direct the production of the protective extracellular matrix. Consistent with this idea, uncoupling of oxidative phosphorylation by CCCP, which mimics hypoxia [52], caused hyper-production of extracellular material in biofilms and higher biofilm stability towards degrading enzymes. Ccr4, while not absolutely required for the cells to execute a transcriptional response to hypoxia, had nevertheless a significant effect on a subset of genes regulated by hypoxia [52]. Our data indicates that post-transcriptional regulation of gene expression by Ccr4 might serve to adjust biofilm metabolism and maturation in response to the harsh hypoxic environment, by impacting on mitochondrial activity and on the expression of cell wall and matrix related genes. Consistent with this proposition, Sellam *et al* have shown that Ccr4 has a role in cell wall gene expression in hypoxia [52].

**Fig 9:**
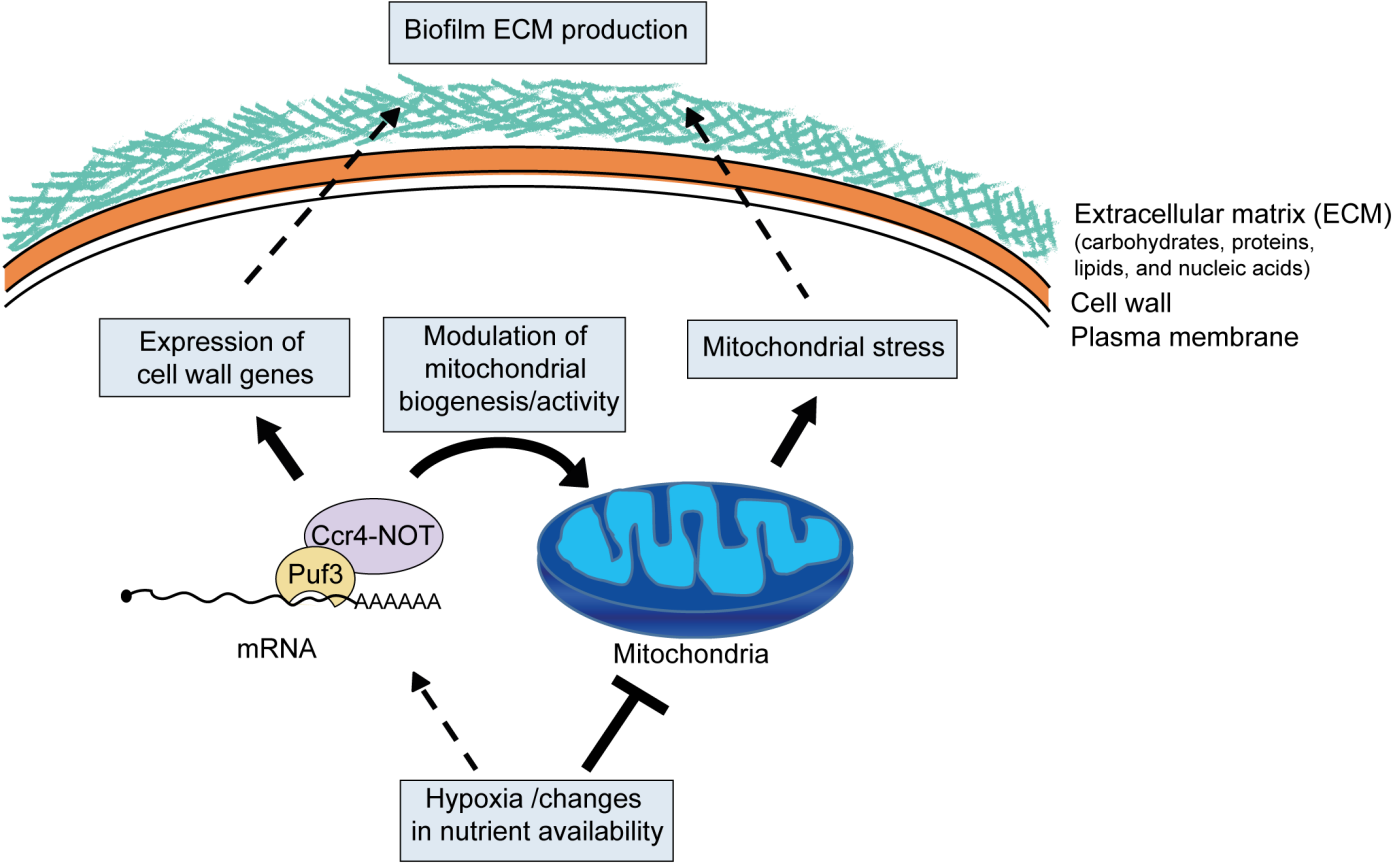
Model for the role of posttranscriptional gene regulation and mitochondrial activity in biofilm matrix production and stress protection. As the biofilms mature, environmental changes, such as hypoxia and nutrients, lead to lowering of mitochondrial activity. Lower mitochondrial activity might be sensed as a stress signal and drive the production of protective extracellular matrix. Cell wall integrity pathways are known to function in matrix production [15,16], and mitochondrial function has been linked to pathways of fungal cell wall integrity (reviewed in [27]), thus providing a plausible mechanism of mitochondrial control over biofilm matrix production. Mitochondrial dysfunction could also lead to weaker cell walls and cell lysis, further contributing to extracellular matrix deposition. Posttranscriptional regulators, such as the Ccr4-NOT mRNA deadenylase and Puf3, coordinate biofilm maturation pathways by responding to nutrients and hypoxia to adjust mitochondrial biogenesis, as well as the expression of genes needed for biofilm matrix production.

Based on the data presented here, we propose a model in which metabolic and mitochondrial reprogramming in biofilms drive the pathways of biofilm maturation (Fig 9). In this model, changes to mitochondrial activity and biogenesis in the biofilm environment, possibly due to hypoxia, constitute the signal that triggers activation of protective mechanisms. This ultimately leads to extracellular matrix accumulation, potentially through the cross-talk between mitochondrial function and the pathways of cell wall biogenesis and overall cell stability (reviewed in [27]). Ccr4 is involved in the response to hypoxia, and it orchestrates biofilm maturation by adjusting the expression of cell wall genes with roles in matrix production, as well as by regulating mitochondrial biogenesis and activity. In conclusion, we suggest that the interface between metabolic and developmental restructuring in biofilms has important consequences for matrix production, a phenotype that is implicated in both antifungal and immune resistance of the biofilm growth mode. This should be considered in the context of antifungal strategies that target metabolic regulators.

## Materials and Methods

### Fungal strains and growth conditions

The open reading frame (ORF) encoding the *C*. *albicans* Puf3 (orf19.1795, C4_05370W) was deleted in the BWP17 strain background, using *URA3* and *ARG4* as selection markers. The complemented strain was made by cloning the *PUF3* ORF plus promoter and terminator regions into the plasmid pDDB78, and this plasmid was integrated into the mutant genome by directing it to the *HIS1* locus. We noticed that the sequence of *PUF3* in the BWP17 strain background somewhat differs from the sequence in the Candida Genome Database. There are deletions of 6 nucleotides at two positions: 1265–1270 and 1593–1598 in 2790 nucleotide long *PUF3* ORF. This affects two repeats: two amino acids P and G are removed from a 4 PG repeat (i.e. leaving 3 PG repeats: PGPGPG), and NN in a 9 N repeat, thereby converting it to a 7 N repeat (NNNNNNN). These two deletions are outside of the PUM domain and do not affect Puf3 function, as shown by complementation of mutant phenotype in Fig 3.

To generate *Candida MET3p* repressible strains, one allele of *COX23* and *MRPL25* were placed under the control of the *MET3* gene promoter by genomic integration via homologous recombination of the *HIS1-MET3p* cassette amplified from the template pFA–HIS1–MET3p plasmid provided by Jürgen Wendland [60]. To generate strains containing mutant forms of the Puf3-binding sites in the 3′ UTR of *COX23*, we first synthesized the whole 2238 bp long cassettes of *MET3p-COX23* ORF containing wild type, core and—C mutant 3′ UTR (GenScript) and cloned these cassettes in pDDB78 plasmid and integrated them into the *HIS1* locus of wild type strain of BWP17 background. All primers used for strain construction are listed in S1 Table. The *C*. *albicans ccr4* and *pop2* mutant strains were described in [29]. For most experiments the wild type control was strain DAY185, which is a fully prototrophic strain derived from BWP17 by re-integration of the *URA3*, *ARG4* and *HIS1* markers. The *S*. *cerevisiae puf3Δ* strain in the *rpb1-1* background and the corresponding control strain were gifts from Wendy Olivas and are described in [36].

Generally, strains were grown in YPD (1% yeast extract, 2% peptone, 2% glucose) medium, supplemented with 80 μg/ml of uridine for *C*. *albicans*. Standard growth temperature was 30°C. For analysis of sensitivity of *puf3Δ/Δ* on different carbon source, ten-fold serially diluted cultures of all strains, were spotted on YP plates containing 2% glucose (YPD), 3% glycerol (YPG) or 3% lactate (YPL) and photographed after 2 days of growth. For sensitivity to CCCP, ten-fold serial dilutions of cultures of wild type and mutant strain were spotted on YPG plates with or without CCCP at the concentrations indicated in Fig 3.

For mRNA decay experiments of *Candida* strains (Fig 4), wild type and *puf3Δ/Δ* strains were first grown overnight and then diluted to OD_600_ = 0.1 and grown further for 4–5 hours in non-inducing YPD or YPL medium at 30°C. To induce expression from *MET3p*, the cultures were washed once with PBS and transferred into synthetic medium lacking methionine and cysteine followed by incubation for 10 min at 30°C. After incubation, the time = 0 min sample was collected, then methionine and cysteine were added to the remaining cultures to repress transcription from *MET3p* (2.5 mM methionine and 1mM cysteine). Samples were collected at indicated time points, flash frozen on dry ice and stored in -80°C until ready for RNA isolation. The experiment was performed 3–4 independent times, assaying one wild type and one mutant culture each time. The experiments with the mutated Puf3 binding site in the *COX23* 3′ UTR were performed twice independently.

For decay experiment of *S*. *cerevisiae* strains (Fig 5), wild type and *puf3Δ* mutant were grown overnight in YPD or YPL medium at 24°C and next day cultures were diluted and grown for few hours until log phase (OD^600^ ~ 0.8). To repress transcription, cultures were quickly centrifuged, and then transferred to medium preheated to 37°C. Samples were collected at indicated time points, flash frozen on dry ice and stored in -80°C prior to RNA isolation.

For gene expression analysis in *C*. *albicans* biofilms (Figs 6A, 7D and 8A), biofilms were grown in 6 well microtiter plates in Spider medium for 48 hours in a 37°C incubator with shaking at 75 rpm. Cell pellets were harvested and stored at -80°C until use. RPMI 1640 buffered with MOPS (RPMI-MOPS, pH = 7.2, for a detailed formula refer to CLSI guideline M27-A3) and Spider medium (1% nutrient broth, 1% D-mannitol, 2 g K_2_HPO_4,_ pH = 7.2) were used for *C*. *albicans* biofilm growth. Biofilm growth in YNB media was performed essentially as described [61].

Mitochondrial morphology in Fig 3D was analyzed by staining log-phase cultures of the indicated *C*. *albicans* strains with 1 μM MitoTracker Red CMXRos (Life Technologies, M7512) for 30 min in the dark at 30°C. Cells were washed with fresh medium, mounted on glass slides and observed with an Olympus BX60 fluorescence microscope. Images were taken with a 100x objective and analyzed using Spot Advanced Software (http://www.spotimaging.com/software/).

### Quantitative PCR analysis of mRNA decay and steady state mRNA levels

For quantitative PCR (qPCR) analysis of all samples, total RNAs were extracted using the hot-phenol method followed by DNase I (Ambion) treatment to remove contaminating genomic DNA. Reverse transcription reaction was performed with Superscript III (Invitrogen) using 800 ng of total RNA and 200 ng of mammalian RNA as a “spike-in” control. qPCR was performed using the Fast-Start universal SYBR Green Master (Roche) on the LightCycler 480 (Roche). The expression levels of the transcripts were normalized to the level of the *SCR1* gene transcribed by RNA polymerase III. Analysis of qPCR data was performed using LinReg [62,63]. The primers used for qPCR analysis are listed in S2 Table.

### ePAT assays

The ePAT assays were performed using RNA extracted from *S*. *cerevisiae* cultures grown in either glucose or lactate as the carbon source. The assay was performed as described in [46]. Primers are shown in S2 Table.

### Biofilm quantitative assays

Biofilm formation of *C*. *albicans* wild type and mutant strains was quantitatively assessed by crystal violet staining, as described in our previous study [64]. The growth medium was Spider or RPMI in 96-well microplates at 37°C. Following 90 min adhesion and washing of non-adherent cells, biofilms were grown for 48 h, with fresh medium added at 24 h. Negative control were wells contained medium-only. Alternatively, *C*. *albicans* biofilms were grown in 6 well plates under the same conditions and total biofilm biomass from three wells for each strain was determined by dry weight measurement. For the XTT reduction assay in Fig 6D, biofilms were grown in RPMI-MOPS for 48 h in 96-well plates, XTT added at 0.5 mg/ml, and reading made at OD_492_ following incubation in the dark for 2 h.

### Biofilm qualitative assay

Scanning electron microscopy was used for biofilm qualitative studies essentially as previously described [64]. Biofilms were formed on serum-coated silicone disks and grown for 48 h at 37°C with shaking at 75 rpm. Samples were viewed under a Hitachi S570 scanning electron microscope.

### Zymolyase sensitivity assays

Biofilms were grown with RPMI-MOPS in 96 well plates for 24 hours, and zymolyase assays performed essentially as described [15], with the exception that crystal violet staining was used for quantification. Zymolyase–20T, MP Biomedicals was used.

### Quantitative analysis of *C*. *albicans* biofilm matrix

Individual *C*. *albicans* biofilm components, including biofilm cells and extracellular polymer matrix (ECM), were isolated from 48 h mature biofilms grown in Spider medium, following the method by Taff *et al* [15]. Total ECM was then extracted and quantified relative to total biofilm consisting of cells and extracellular material using a method modified from McCourtie and Douglas [65]. β-1,3 glucan in the biofilm ECM was determined quantitatively using the Glucatell Endpoint Kit (DKSH).

### Statistical analysis

Statistical analysis was performed on the biological repeats. Where multiple technical repeats were performed for a given biological repeat, the values of the technical repeats were averaged to give the data point for that particular biological sample. Statistical significance was calculated in GraphPad Prism using the Student’s t-test.

### RNA seq and bioinformatics analysis

The PAT-seq data utilized for 3′ analysis here is available in the NCBI Short Read Archive (accession number SRP056994). The tracks suitable for upload of all adenylation-sites we identified in the *C*. *albicans* transcriptome into gbrowse (CGD) the follow steps can be taken: 1) open gbrowse and set genome data source to A21 assembly; 2) go to ‘select custom tracks’; 3) copy and paste the web link within verma-gaur et al., PAS-5.wig; 4) go back to browser and click on the little spanner symbol to change the track-height to 80. Note, use the down cursor rather than mouse; 5) Do the same for forward and reverse tracks. The *S*. *cerevisiae* data is drawn from [41], and is available from GEO accession GSE53461 and for interactive viewing at http://rnasystems.erc.monash.edu/.

For PAT-seq the *C*. *albicans* strain was grown in YPD+Uridine to mid log phase at 30°C, and the PAT-seq experiment was performed exactly as previously described [41], except that a template oligonucleotide compatible with SOLiD sequencing [/5BioTEG/CTGCTGTACGGCCAAGGCGTTTTTTTTTTTT] was used to append the 3’ tag, and SOLiD compatible 5’ linkers were ligated to the 5’ end. PAT-seq cDNA was input into 16 cycles of amplification with a SOLiD universal sequencing primer [CCACTACGCCTCCGCTTTCCTCTCTATGGGCAGTCGGAGAT] and SOLiD Barcoding primers (Life Technologies). PAT-seq libraries were sequenced on a *SOLiD* 5500xl instrument according to the manufacturer’s instructions at the Gandel Charitable Trust Sequencing Centre (Monash University). The data were mapped to the reference genome: *C*. *albicans* SC5314 assembly 21; using the tail-tools pipeline version 0.31 and nesoni version 0.117) http://rnasystems.erc.monash.edu/. Figures were generated in R and Illustrator. The Venn diagram in Fig 3 was generated using US DOE Venn Diagram Plotter software (http://omics.pnl.gov/software/venn-diagram-plotter), and we acknowledge PNNL and the OMICS.PNL.GOV website. For comparative analyses, the list of *S*. *cerevisiae* and *C*. *albicans* orthologs was obtained from the Candida Genome Database (candidagenome.org). For the analysis of the relationship between 3′ UTR length and mitochondrial function, the list of genes annotated to GO mitochondria was from Amigo 2, GO:0005739.

## Supporting Information

S1 Fig Lack of conservation of 3′ UTR length in putative Puf3 targets in *S*. *cerevisiae* and *C*. *albicans*. The data is the same as in Fig 2C, and represents the comparison between the 3′ UTR lengths of the 3552 orthologous genes between *C*. *albicans* and *S*. *cerevisiae*. Here, the putative Puf3 targets conserved between the two yeasts are indicated in red.
(TIF)

S2 Fig The effects of the transcriptional inhibitors 1,10 phenantroline and thiolutin on mRNA stability in *C*. *albicans*. (A) Decay of indicated mRNAs in the wild type *Candida* strain following transcriptional repression by 1,10 phenantroline at 1 mg/ml. The levels of mRNAs were measured by qPCR. The decay curves and half-life (T1/2) were calculated using the nonlinear regression (curve fit) method using the exponential, one phase decay equation. (B) Decay of indicated mRNAs in wild type *Candida* strain following transcription repression by thiolutin at final concentration of 20 μg/ml. The fold expression of indicated RNAs was measured by qPCR analysis and the decay curves and half-life (T1/2) were measured as in (A). The half-life of most of the RNA tested was longer than expected, compared to what has been measured for these transcripts in the model yeast *Saccharomyces cerevisiae*. For example, Munchel *et al* measured half lives of 25 min for *COX23* and 18 min for *TOM40* and *COX11* [67], Holstege *et al* measured 12 min for *COX11* and 17 min for *TOM40* [68], while Geisberg *et al* measured 21 min for *COX11* and 31 min for *TOM40* [69]. These results suggested that thiolutin treatment might have an indirect, stabilizing effect on mRNA half-life. (C) Transcriptional repression by thiolutin treatment had a strong effect on the mRNA half-life. To test this, log phase cultures of wild type strain with methionine-repressible *MET3p*-*SAM50* gene were treated with methionine and cysteine (Met/Cys) and/or thiolutin at the final concentration of 20 μg/ml. RNA levels were measured by qPCR analysis and half-life was calculated as in (A). *SAM50* mRNA was rapidly decayed (half-life = 3.4 min) following transcriptional shut down by Met/Cys in the absence of thiolutin. However, the half-life was much longer in the presence of thiolutin.
(TIF)

S3 Fig Validation of the *MET*-off experimental system for mRNA decay analysis in *C*. *albicans*. (A) A cartoon depicting the location of the primers used for specific amplification of the *MET3p*-driven *COX23* and *MRPL25* genes (same as Fig 4A). (B) qPCR analysis showing the expression levels of *MET3p*-driven *COX23* and *MRPL25* in wild type and *puf3Δ/Δ* mutant. The expression levels of *COX23* and *MRPL25* were induced for 10 minutes in synthetic media without methionine and cysteine (Met/Cys). The analysis by qPCR using the primers depicted in (A) showed expected gene induction and repression after addition of Met/Cys.
(TIF)

S4 Fig Growth properties of the *C*. *albicans MET3p-COX23* and *MET3p-MRPL25* strains. Indicated wild type and *puf3Δ/Δ* mutant were inoculated at OD_600_ = 0.1 in YPD (A) or YPL (B) media and growth rates were measured by taking OD_600_ at indicated time intervals. The data are represented as mean and standard error of 4 independently grown cultures for each strain.
(TIF)

S5 Fig Decay of *S*. *cerevisiae* transcripts encoding mitochondrial proteins that lack Puf3 binding sites. Decay of the indicated mRNAs was measured in the wild type and *puf3Δ* strains grown in glucose (A) or lactate (B) following transcriptional repression at 37°C, using the RNA samples described in Fig 5. The data are shown as the average and standard error of 2–3 biological replicates.
(TIF)

S6 Fig Analysis of alternative 3′ UTRs in response to carbon source in *S*. *cerevisiae*. (A) Cartoon depicting the distal and proximal polyadenylation sites and the extension poly(A) test (ePAT) assay. (B) Results of the ePAT assay using samples of wild type and *puf3Δ* mutant RNA grown in glucose or lactate as the carbon source. In lactate, *TOM70* and *OM14* display an mRNA with a shorter 3a UTR in both yeast strains.
(TIF)

S7 Fig Effects of *CCR4* and *PUF3* on biofilm gene expression. Shown are additionally genes tested in biofilm samples derived from wild type, *ccr4Δ/Δ* and *puf3Δ/Δ* mutant biofilms shown in Fig 6A. Error bars are ± standard errors of the average of 3 biological replicates.
(TIF)

S8 Fig Biofilm formation by the *puf3Δ/Δ* mutant. (A) Scanning electron micrographs of mature biofilms (48 h) formed on silicone disks in Spider medium. The assay was repeated twice. (B) Biomass of 48 h biofilms grown in 96-well microplates with Spider medium was quantified using the crystal violet staining assay. Results were calculated from three independent repeats in triplicate. The error bar represents the standard error. No difference was found between biofilms formed by the *puf3Δ/Δ* mutant strain and the complemented strain *puf3Δ/Δ* + *PUF3* (*p* = 0.37).
(TIF)

S9 Fig Inactivation of the *CCR4* and *POP2* mRNA deadenylase genes leads to biofilm matrix overproduction regardless of growth medium. Shown are scanning electron micrographs of 48 h biofilms formed on silicone disks in either RPMI-MOPS or YNB media. Biofilm extracellular matrix is indicated with black arrows. Scale bar = 10 μm.
(TIF)

S10 Fig Effects of CCCP treatment on biofilm growth. Quantitative determination of biofilm formation by wild type *C*. *albicans* in the presence of CCCP. Crystal violet staining assay was performed for biofilms formed in 96-well microplates with RPMI-MOPS. A reduction of the total biofilm biomass was observed for the biofilm grown with CCCP (20 μM). However, this inhibition does not prevent *C*. *albicans* from growing into complex multi-cellular biofilm structure (see SEM in Fig 8B). Results were calculated from three biological repeats in technical triplicates. Error bar represents the standard error. ***: *p* <0.001.
(TIF)

S1 Dataset Analyses of genes down-regulated in *C*. *albicans* biofilms. The biofilm dataset was from [14].
(XLSX)

S2 Dataset Bioinformatic analyses of 3′ UTRs and Puf3 binding sites. The *C*. *albicans* PATseq data is from this study and described in Materials and Methods. The *S*. *cerevisiae* PAT-seq data is from [41].
(XLSX)

S3 Dataset Gene ontology analysis relative to 3′ UTR length. (XLSX)

S4 Dataset Functional analyses of the *C*. *albicans* mitochondrial mRNA regulon of putative Puf3 targets. (XLSX)

S1 Table Primers used for strain construction. (DOCX)

S2 Table Primers used for qPCR and ePAT assays. (DOCX)
