# Supplementary figures and images for: Integration of Posttranscriptional Gene Networks into Metabolic Adaptation and Biofilm Maturation in Candida albicans
Source: PLoS Genet. 2015 Oct 16;11(10):e1005590. doi: 10.1371/journal.pgen.1005590 (PMC4608769; doi:10.1371/journal.pgen.1005590)

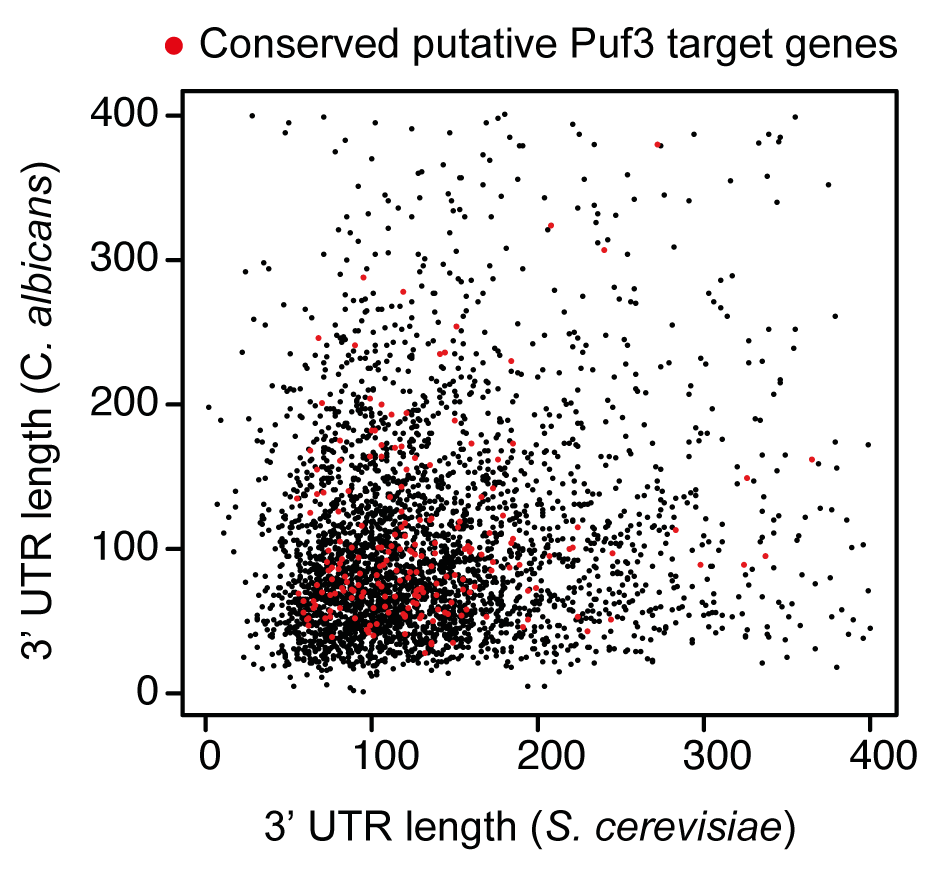

Supplement: S1 Fig — The data is the same as in Fig 2C, and represents the comparison between the 3′ UTR lengths of the 3552 orthologous genes between C. albicans and S. cerevisiae. Here, the putative Puf3 targets conserved between the two yeasts are indicated in red. (TIF) [file pgen.1005590.s001.tif]

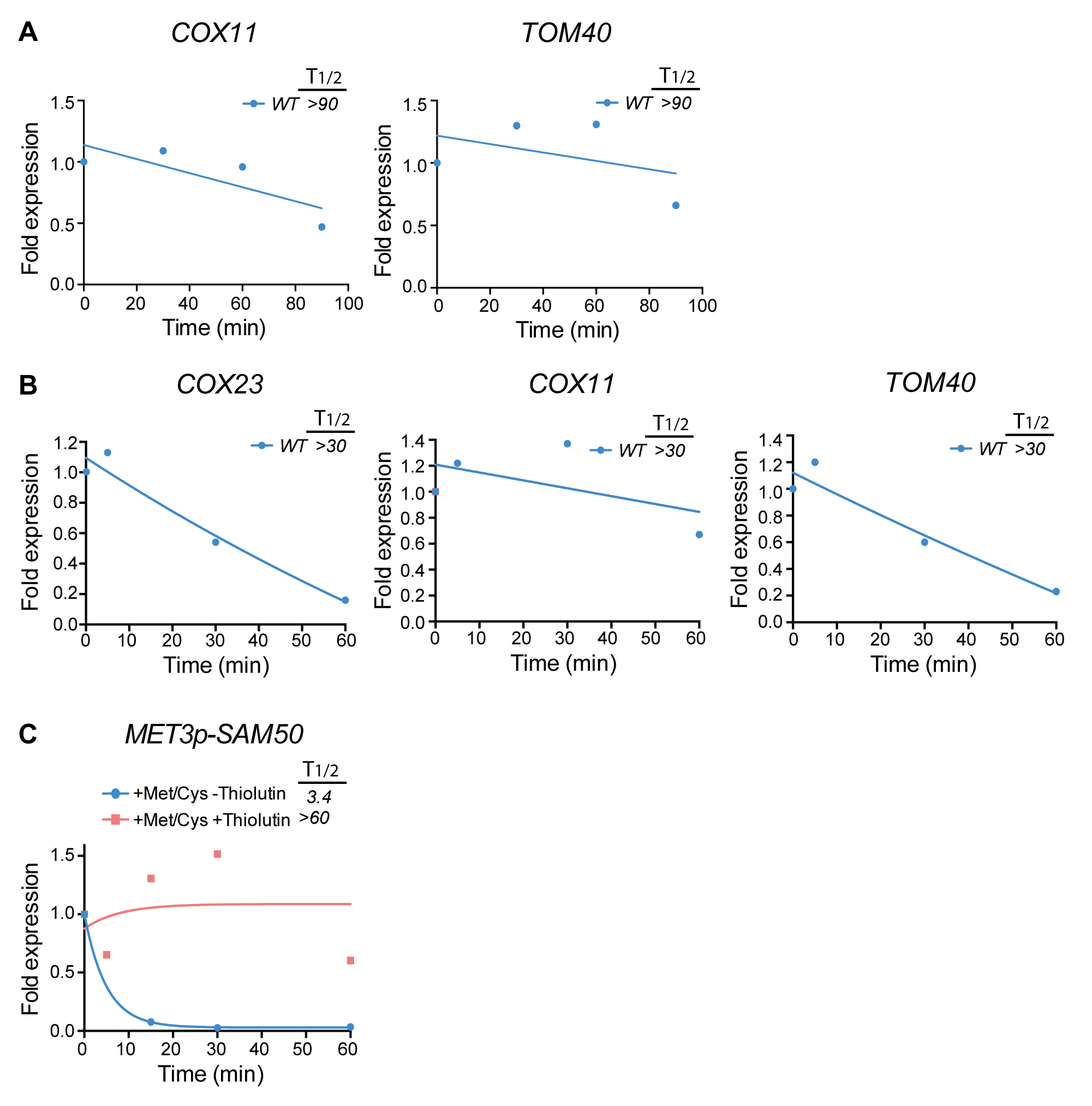

Supplement: S2 Fig — (A) Decay of indicated mRNAs in the wild type Candida strain following transcriptional repression by 1,10 phenantroline at 1 mg/ml. The levels of mRNAs were measured by qPCR. The decay curves and half-life (T1/2) were calculated using the nonlinear regression (curve fit) method using the exponential, one phase decay equation. (B) Decay of indicated mRNAs in wild type Candida strain following transcription repression by thiolutin at final concentration of 20 μg/ml. The fold expression of indicated RNAs was measured by qPCR analysis and the decay curves and half-life (T1/2) were measured as in (A). The half-life of most of the RNA tested was longer than expected, compared to what has been measured for these transcripts in the model yeast Saccharomyces cerevisiae. For example, Munchel et al measured half lives of 25 min for COX23 and 18 min for TOM40 and COX11 [67], Holstege et al measured 12 min for COX11 and 17 min for TOM40 [68], while Geisberg et al measured 21 min for COX11 and 31 min for TOM40 [69]. These results suggested that thiolutin treatment might have an indirect, stabilizing effect on mRNA half-life. (C) Transcriptional repression by thiolutin treatment had a strong effect on the mRNA half-life. To test this, log phase cultures of wild type strain with methionine-repressible MET3p-SAM50 gene were treated with methionine and cysteine (Met/Cys) and/or thiolutin at the final concentration of 20 μg/ml. RNA levels were measured by qPCR analysis and half-life was calculated as in (A). SAM50 mRNA was rapidly decayed (half-life = 3.4 min) following transcriptional shut down by Met/Cys in the absence of thiolutin. However, the half-life was much longer in the presence of thiolutin. (TIF) [file pgen.1005590.s002.tif]

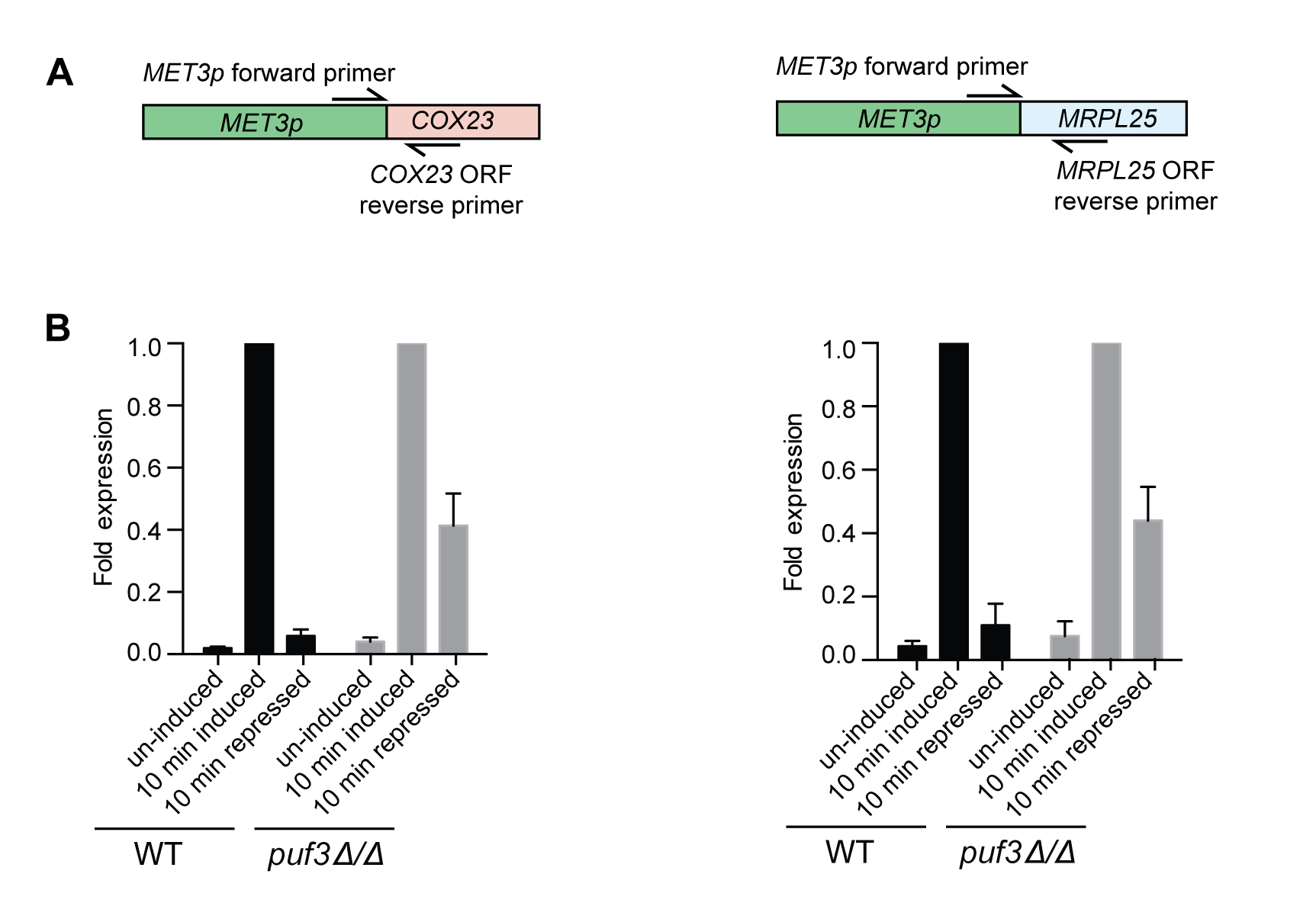

Supplement: S3 Fig — (A) A cartoon depicting the location of the primers used for specific amplification of the MET3p-driven COX23 and MRPL25 genes (same as Fig 4A). (B) qPCR analysis showing the expression levels of MET3p-driven COX23 and MRPL25 in wild type and puf3Δ/Δ mutant. The expression levels of COX23 and MRPL25 were induced for 10 minutes in synthetic media without methionine and cysteine (Met/Cys). The analysis by qPCR using the primers depicted in (A) showed expected gene induction and repression after addition of Met/Cys. (TIF) [file pgen.1005590.s003.tif]

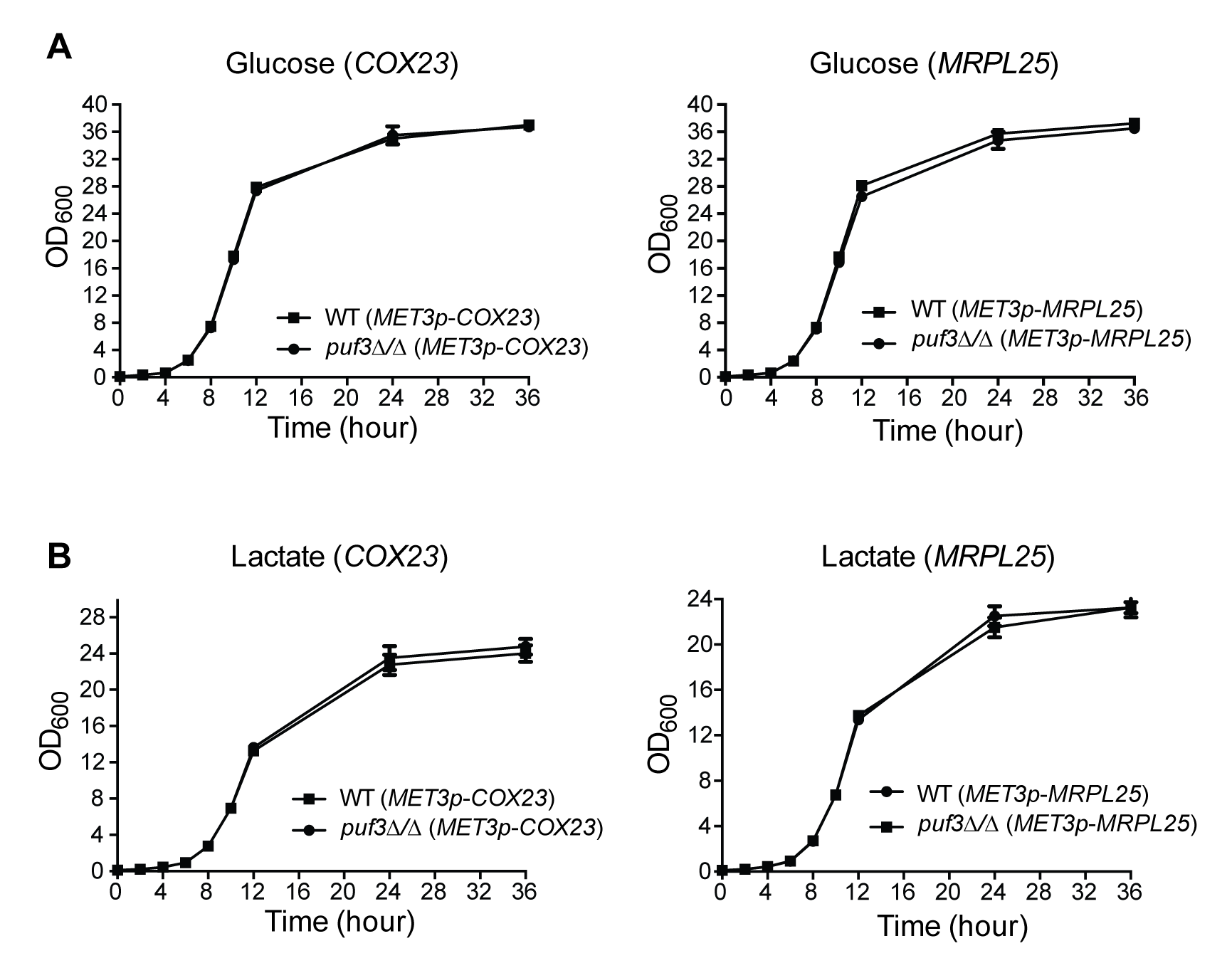

Supplement: S4 Fig — Indicated wild type and puf3Δ/Δ mutant were inoculated at OD600 = 0.1 in YPD (A) or YPL (B) media and growth rates were measured by taking OD600 at indicated time intervals. The data are represented as mean and standard error of 4 independently grown cultures for each strain. (TIF) [file pgen.1005590.s004.tif]

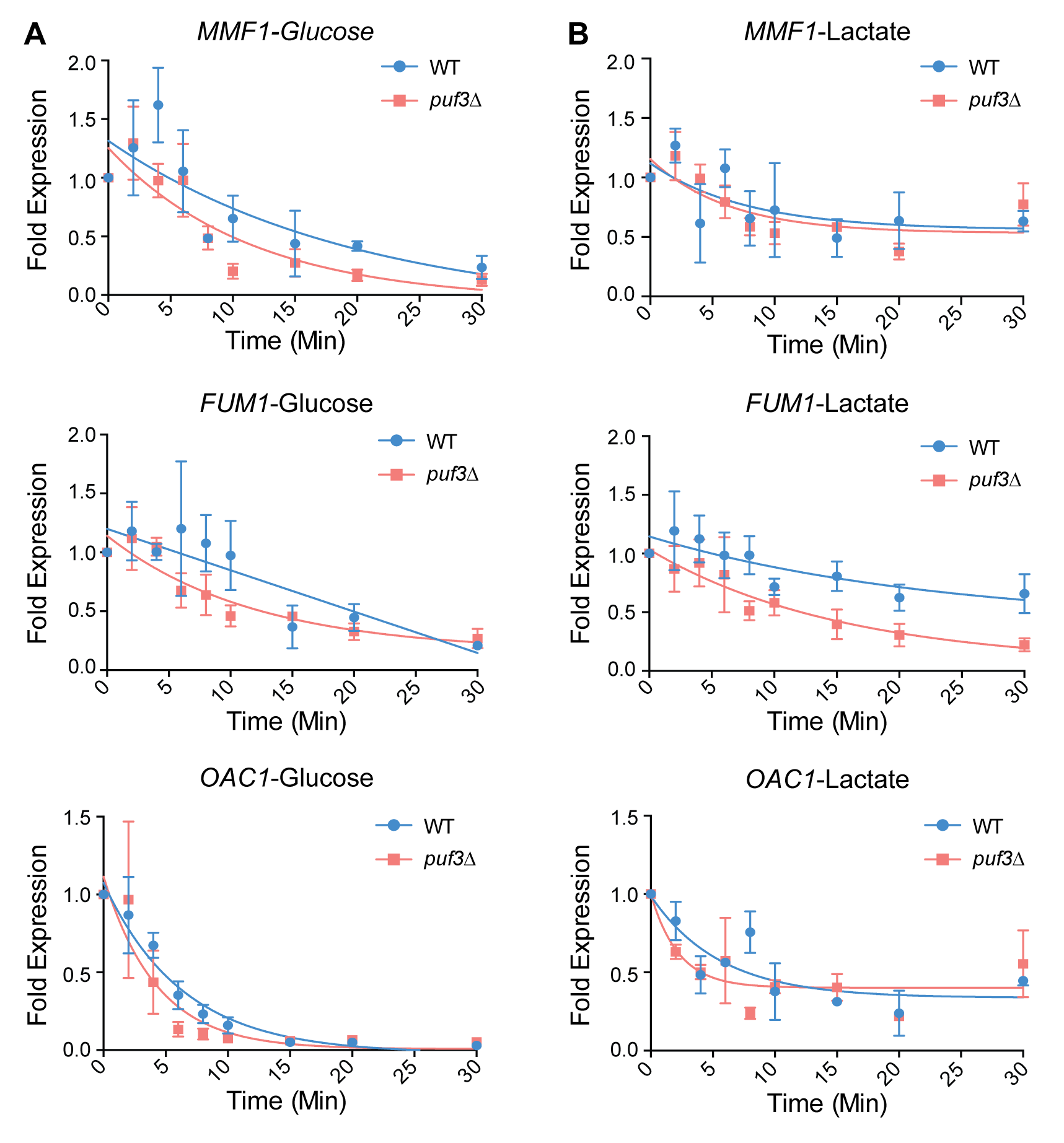

Supplement: S5 Fig — Decay of the indicated mRNAs was measured in the wild type and puf3Δ strains grown in glucose (A) or lactate (B) following transcriptional repression at 37°C, using the RNA samples described in Fig 5. The data are shown as the average and standard error of 2–3 biological replicates. (TIF) [file pgen.1005590.s005.tif]

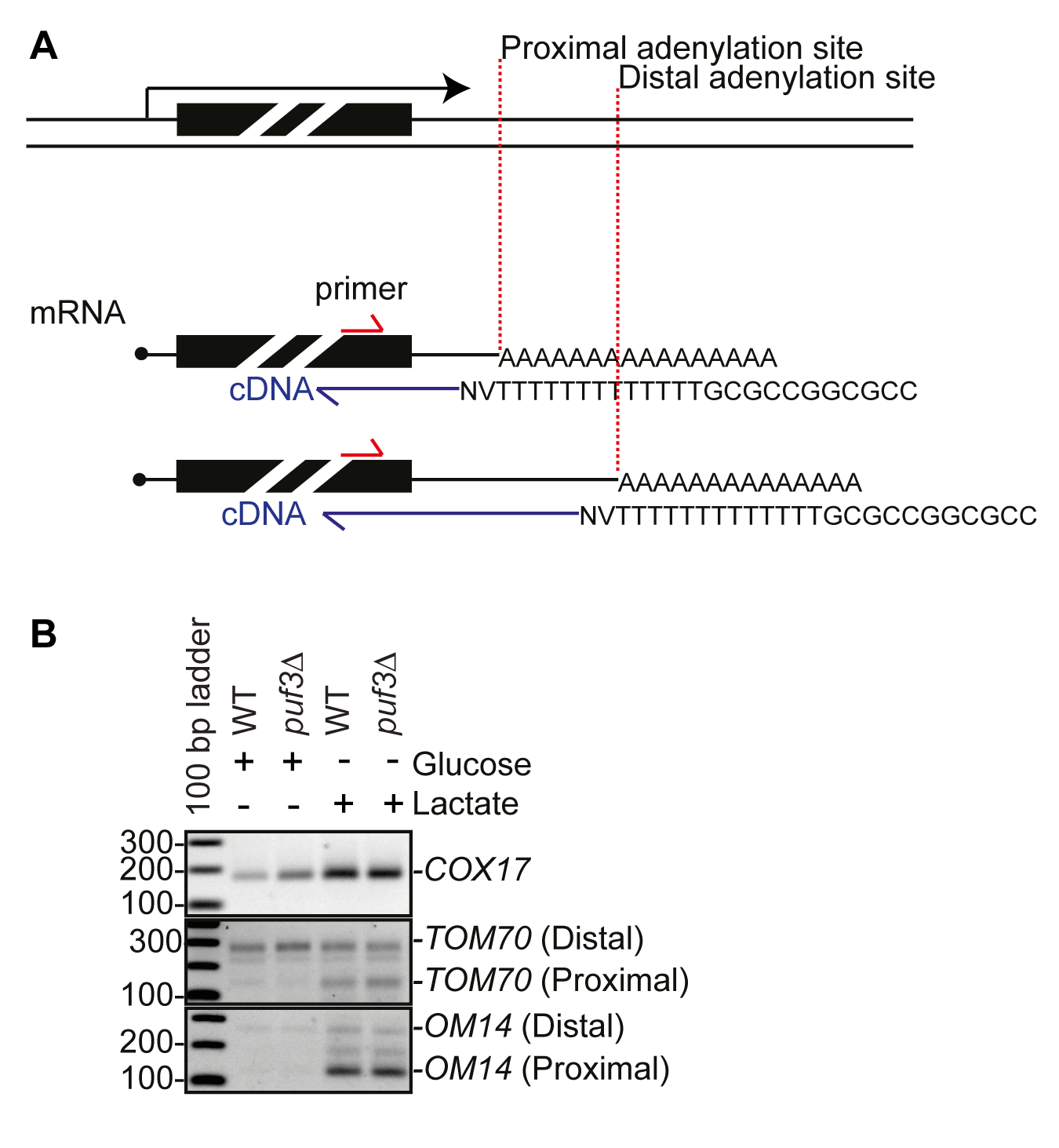

Supplement: S6 Fig — (A) Cartoon depicting the distal and proximal polyadenylation sites and the extension poly(A) test (ePAT) assay. (B) Results of the ePAT assay using samples of wild type and puf3Δ mutant RNA grown in glucose or lactate as the carbon source. In lactate, TOM70 and OM14 display an mRNA with a shorter 3a UTR in both yeast strains. (TIF) [file pgen.1005590.s006.tif]

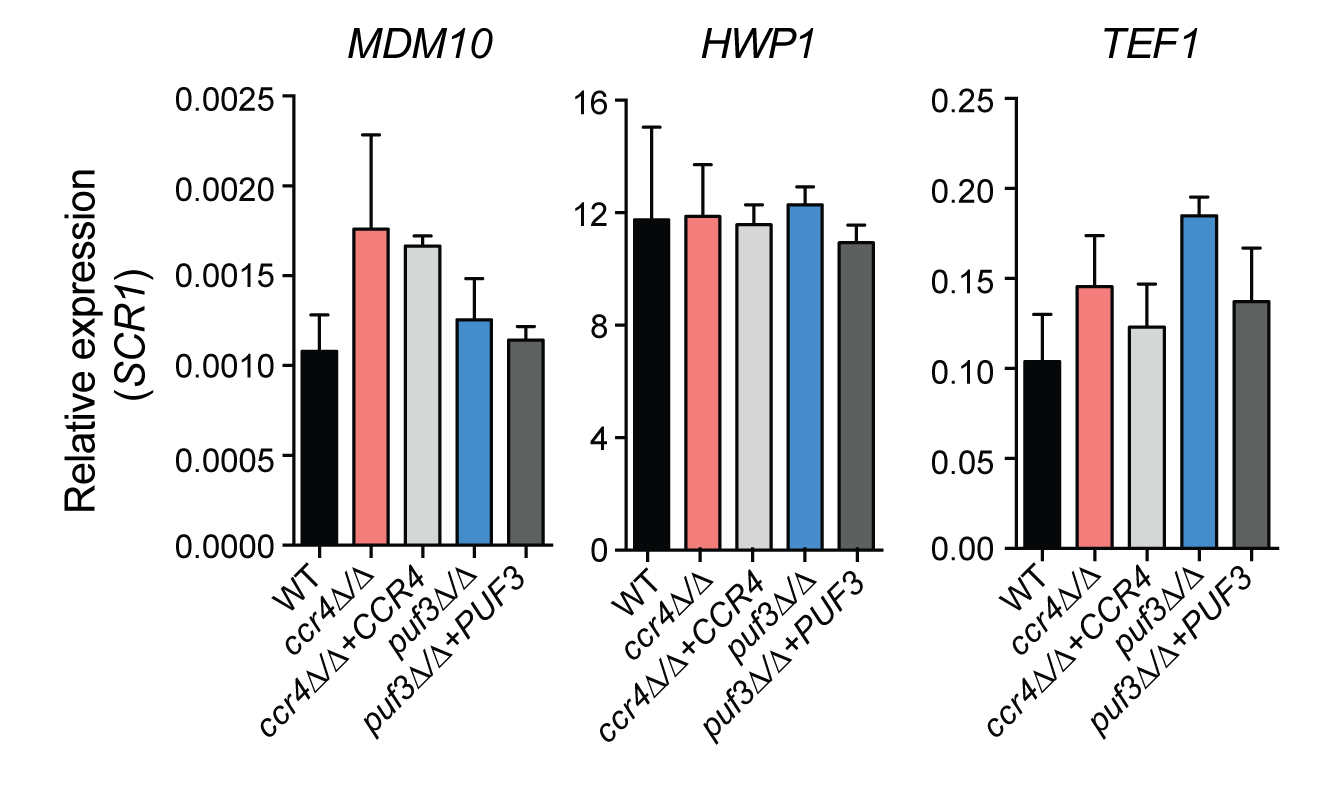

Supplement: S7 Fig — Shown are additionally genes tested in biofilm samples derived from wild type, ccr4Δ/Δ and puf3Δ/Δ mutant biofilms shown in Fig 6A. Error bars are ± standard errors of the average of 3 biological replicates. (TIF) [file pgen.1005590.s007.tif]

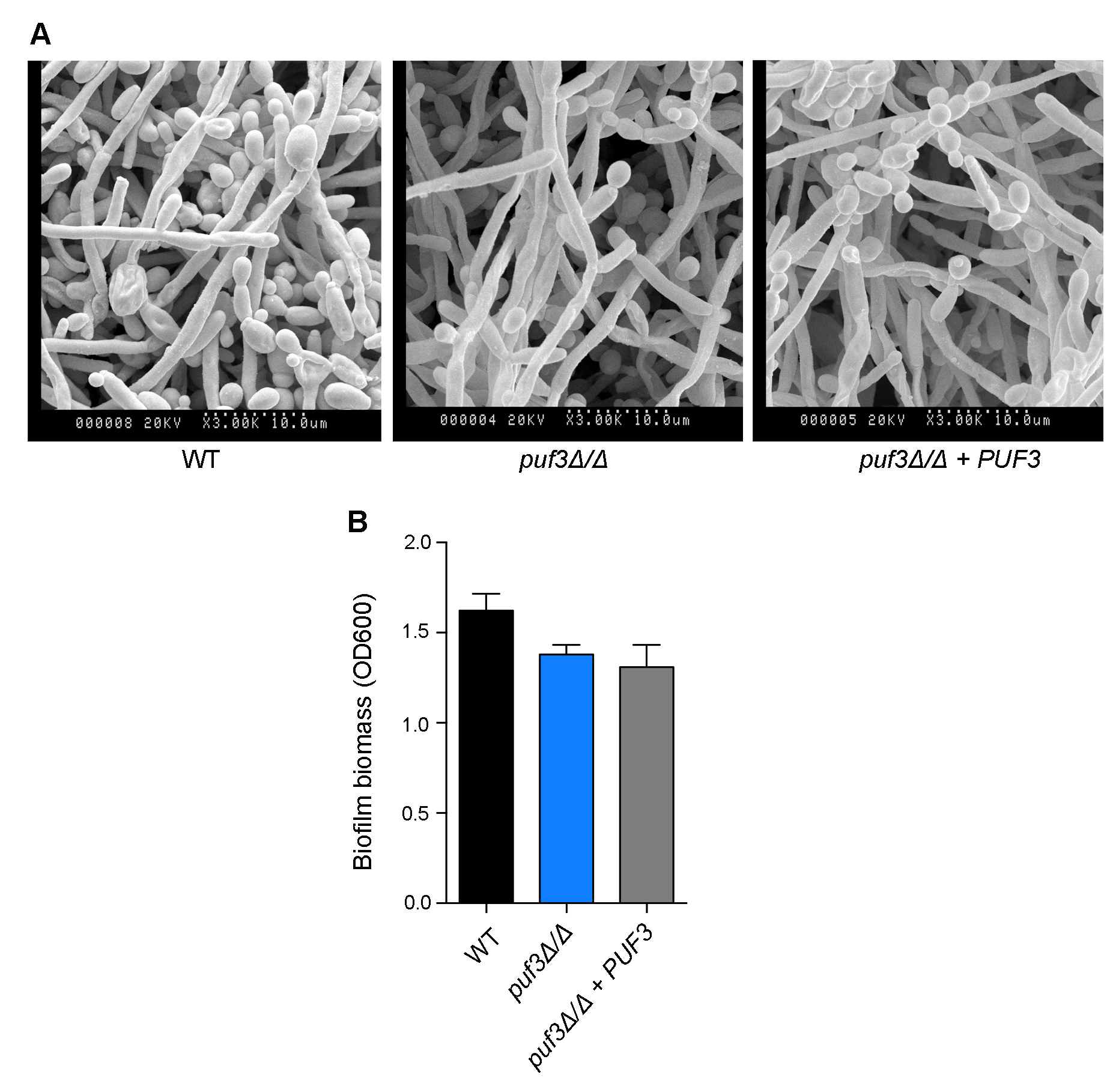

Supplement: S8 Fig — (A) Scanning electron micrographs of mature biofilms (48 h) formed on silicone disks in Spider medium. The assay was repeated twice. (B) Biomass of 48 h biofilms grown in 96-well microplates with Spider medium was quantified using the crystal violet staining assay. Results were calculated from three independent repeats in triplicate. The error bar represents the standard error. No difference was found between biofilms formed by the puf3Δ/Δ mutant strain and the complemented strain puf3Δ/Δ + PUF3 (p = 0.37). (TIF) [file pgen.1005590.s008.tif]

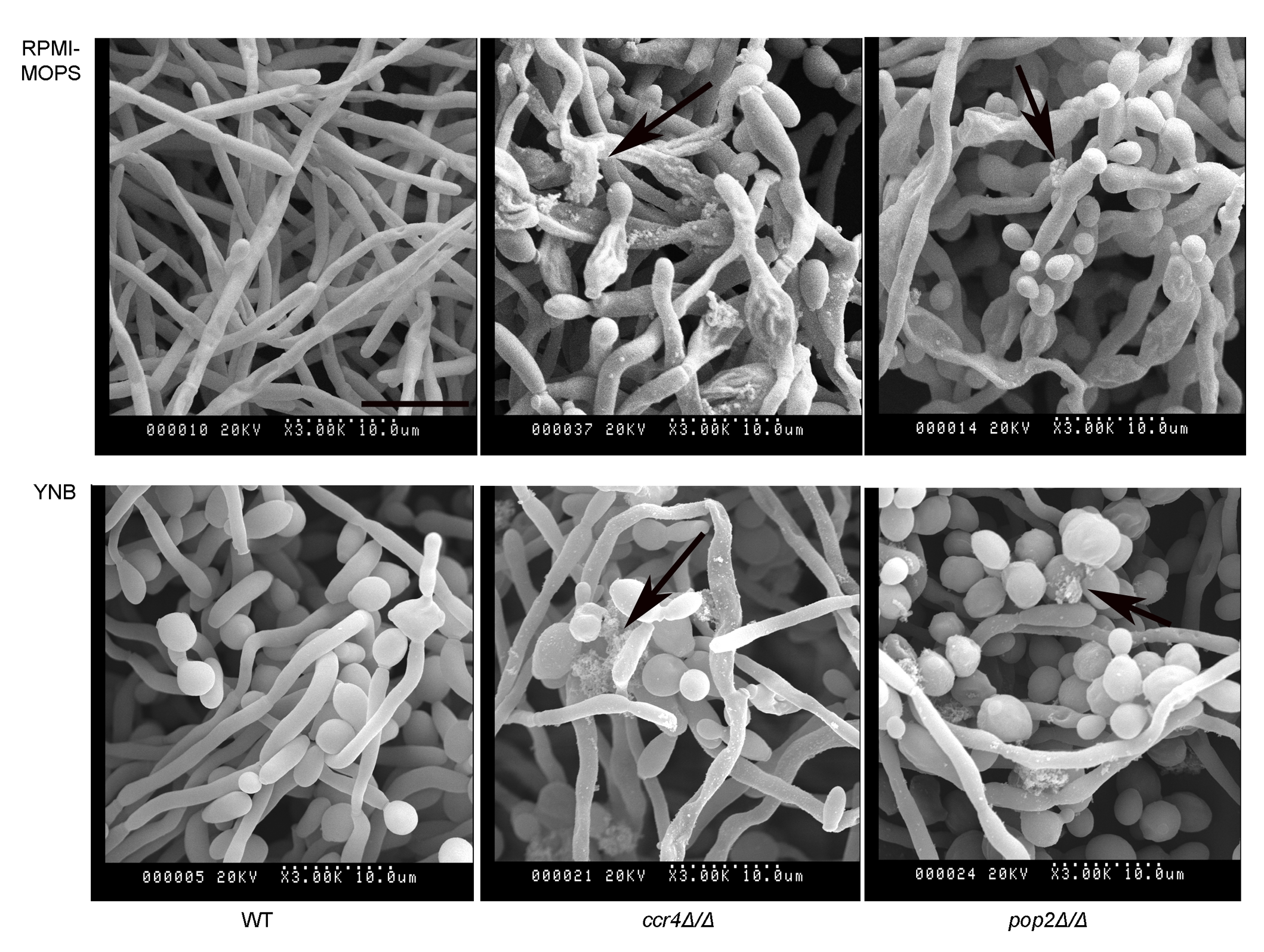

Supplement: S9 Fig — Shown are scanning electron micrographs of 48 h biofilms formed on silicone disks in either RPMI-MOPS or YNB media. Biofilm extracellular matrix is indicated with black arrows. Scale bar = 10 μm. (TIF) [file pgen.1005590.s009.tif]

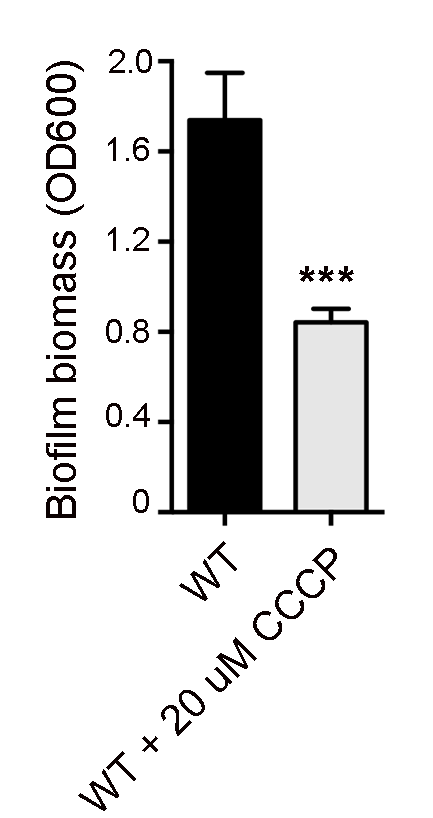

Supplement: S10 Fig — Quantitative determination of biofilm formation by wild type C. albicans in the presence of CCCP. Crystal violet staining assay was performed for biofilms formed in 96-well microplates with RPMI-MOPS. A reduction of the total biofilm biomass was observed for the biofilm grown with CCCP (20 μM). However, this inhibition does not prevent C. albicans from growing into complex multi-cellular biofilm structure (see SEM in Fig 8B). Results were calculated from three biological repeats in technical triplicates. Error bar represents the standard error. ***: p <0.001. (TIF) [file pgen.1005590.s010.tif]
