# Supplementary material for: Integration of Posttranscriptional Gene Networks into Metabolic Adaptation and Biofilm Maturation in Candida albicans
Source: PLoS Genet. 2015 Oct 16;11(10):e1005590. doi: 10.1371/journal.pgen.1005590 (PMC4608769; doi:10.1371/journal.pgen.1005590)
Supplement: S1 Table — (DOCX) [file pgen.1005590.s015.docx]

**S1 Table**: Primers used for strain construction

| Name | Primer sequence |
| --- | --- |
| PUF3 KO (F) | TTAAATTCTTTTTGGTAAAACCACAAACAGAAACAAAAATCCAATTCATTAAACAATCAATTTTTGGTCAAAACATTTTGTGACAAAGAAGACCCCAAATTTTCCCAGTCACGACGTT |
| PUF3 KO (R) | AAAAAAAAAAAACAAGAAAACTATAATAGCTAAAGGGTATTGTAAGCCATTGGGTTGATTGGAAAGTTCTCACCAAATCATTTACCTCCTTTCCCTCTTAGTGGAATTGTGAGCGGATA |
| PUF3 5’ check (F) | CTCGATTTAACCACGTTTCA |
| PUF3 5’ check (R) | GTTGTGGTAGATGCCAACAG |
| PUF3 3’ check (F) | TGATGATGATATTGATGGTG |
| PUF3 3’ check (R) | AATCTCGATAAATCTTCAAC |
| PUF3 complement (F) | TTCACACAGGAAACAGCTATGACCATGATTACGCCAAGCTCAGCGATTGGGATCGGTAAT |
| PUF3 complement (R) | TCGACCATATGGGAGAGCTCCCAACGCGTTGGATGCATAGATGATTGCCAAATAAGTTTA |
| MET3p-COX23 strain generation (F) | CCAATTTTTAGAGCCACTACCGATTTAATTTTTCTTTATTGGGTTGCCAATTTATGTATTTACTTTGTTGGATAGTAAACTGGCCCCCATATTCAAAGCAGAAGCTTCGTACGCTGCAGGTC |
| MET3p-COX23 strain generation (R) | GTTGCTGTTTTGTGGTAGGGGTGGTAGTAGCAGTAGTATTAGTATTCTTTGGAATTATGGGTGTATCTGCTTCCTTTTTAGTTTCAGTAATCTCCGTCAT CATGTTTTCTGGGGAGGGTATTTAC |
| MET3p-MRPL25 strain generation (F) | AATGTAGAAATCTATAGTGCTACCAAGTGTATAGTCGAATAAAAAAAAAGAAAAATTTTTCTCCAAAAAAAAAAGACACACCCCAAACAACAACCAAACCGAAGCTTCGTACGCTGCAGGTC |
| MET3p-MRPL25 strain generation (R) | TGTACTGTGCAAATGGTCGTGGTGGGTATTTGATGAAAAAGTTGTGCAACTTCTGGGGGAGTTTGGCGAATGCCTCTTTGGGAGTGAGTTGGAAAGACAT CATGTTTTCTGGGGAGGGTATTTAC |
| COX23 5’ Check (F) | GACGTCTGATTCTAAAAGTGCTGA |
| COX23 3’ Check (R) | ATAAGCAATCCATAGAAGCCATTC |
| MRPL25 5’ Check (F) | TTGCCGATTTCTCAACTAGAGAGT |
| MRPL25 3’ Check (R) | CTTGGTTTTTGTTTGGGAAAAAT |
| HIS1 Internal Check (R) [1] | CAACGAAATGGCCTCCCCTACCACAG |
| HIS1 Internal Check (F) [1] | GGACGAATTGAAGAAAGCTGGTGCAACCG |

1. Gola S, Martin R, Walther A, Dunkler A, Wendland J (2003) New modules for PCR-based gene targeting in Candida albicans: rapid and efficient gene targeting using 100 bp of flanking homology region. Yeast 20: 1339-1347.
