# Supplementary material for: Integration of Posttranscriptional Gene Networks into Metabolic Adaptation and Biofilm Maturation in Candida albicans
Source: PLoS Genet. 2015 Oct 16;11(10):e1005590. doi: 10.1371/journal.pgen.1005590 (PMC4608769; doi:10.1371/journal.pgen.1005590)
Supplement: S2 Table — (DOCX) [file pgen.1005590.s016.docx]

**S2 Table**: Primers used for qPCR and ePAT

| Name | Primer sequence |
| --- | --- |
| Ca18sRNA (F) | GGATTTACTGAAGACTAACTACTG |
| Ca18sRNA (R) | GAACAACAACCGATCCCTAGT |
| CaSCR1 (F) | TTTAGCATAACCACTGGAGGGAAG |
| CaSCR1 (R) | GAGTTGCAACACTAGATACCGCACT |
| MET3p (F) | GTAAATACCCTCCCCAGAAAACATG |
| CaCOX23-ORF (R) | TCTGATATCAATTTTGTGGGTTGC |
| CaMRPL25-ORF (R) | GCAACAAGTCGTGGATACCAAA |
| CaCOX11 (F) | ACAGCCATCTGTTCCAGACC |
| CaCOX11 (R) | CCACCCAGTACGTTGACAGA |
| CaCOX23 (F) | TGACCCATGTGAAGAGAGTAGA |
| CaCOX23 (R) | CCGACATTCCCTATAAGCATC |
| CaMRPL25 (F) | CCACGACCATTTGCACAGTA |
| CaMRPL25 (R) | TGGAACTTTCTTGGCGTAGG |
| CaTOM40 (F) | ACGGATGGGACAAATCAAAC |
| CaTOM40 (R) | GACTGCCAACTTGGATGACA |
| CaTOM20 (F) | AGGCAAAAGTTGATGCCATT |
| CaTOM20 (R) | TGACAGTTGGTGCTCCCTTA |
| CaPOR1 (F) | GCTGATTCTGGTTTGACTGC |
| CaPOR1 (R) | AACTGGTTCAGCCAATTTCA |
| CaMDM12 (F) | ACCACGGTGATGGGATCAATAA |
| CaMDM12 (R) | TTCATCATCCTCGTCGTCATCC |
| CaMDM10 (F) | ACCCTGCTGTGATTCCCAAA |
| CaMDM10 (R) | TGCTGCTGATAATCCAGGAC |
| CaHWP1 (F) | AATCCTCCTCAACCTGATCAGCCTG |
| CaHWP1 (R) | AGCTGGAGTTGTTGGCTTTTCTGGA |
| CaTEF1 (F) | GAATTCGAAGCCGGTATTTC |
| CaTEF1 (R) | CAGAGATTGGAACGAATGGA |
| CaFKS1 (F) | TGCCGTCTTAATATCGTTGG |
| CaFKS1 (R) | TTTCGGACACTTCCTGACAC |
| CaBGL2 (F) | GCTGCTGAAGCTGAAGGATT |
| CaBGL2 (R) | GTGGAGACGGAAATCTTTGG |
| CaXOG1 (F) | GTTGCTGAAGGTCAATGGAA |
| CaXOG1 (R) | GCAACTGAAATGTGGTCGTT |
| CaPHR1 (F) | CTAATTTGCCACCAACTCCA |
| CaPHR1 (R) | TCGTCAGCAACAACACATTC |
| CaKRE1 (F) | AATCCTTGCGGCAGATAAA |
| CaKRE1 (R) | CCATTGGCATCAGTACCTGT |
| CaALG11 (F) | ATGAACATTTTGGTATTGGAGTGGT |
| CaALG11 (R) | TTTGTAAATTGGGATCAAGATCAGG |
| CaMNN9 (F) | GATTTCAATAATTGGGTCGAAAGTG |
| CaMNN9 (R) | GTCAGCTTTAACCATAACTGCACCT |
| CaVRG4 (F) | TTCATTGAATTTCCCTGCTGATAAT |
| CaVRG4 (R) | TGGTAATTTATTCAATGCTCCAACC |
| CaVAN1 (F) | GTACCCAATGTGTGGAGACCTTTAC |
| CaVAN1 (R) | TTACTTCTCGAGGATCACCATTAGC |
| CaPMR1 (F) | CTGACGGTGTGATTACTGCTAGAGA |
| CaPMR1 (R) | ATGATCCAAGGACAGCAAAATTAAA |
| ScSCR1 (F) | TTCACCGCTGTTAGGGGAGTTTTAT |
| ScSCR1 (R) | CACAATGTGCGAGTAAATCCTGATG |
| ScCOX17 (F) | GGAGTGCGAGGACAAACCTA |
| ScCOX17 (R) | AGCCATAACCCTTCATGCAC |
| ScCOX23 (F) | TGGCACTGCGTTATGGAGTA |
| ScCOX23 (R) | CCCTTCTTGGAAGCAATCAA |
| ScMRPL25 (F) | GATTACGACCGATCCAAGTG |
| ScMRPL25 (R) | TCATTCCCACTGCTGTCTG |
| ScMRP21 (F) | GAGTGACGGGTTTTGGAGAA |
| ScMRP21 (R) | CGCCTGTAACACCCCTAATG |
| ScMRPL11 (F) | CCCTCAACAAGTAGCCAAGC |
| ScMRPL11 (R) | ACGTACGAACCAAACCCAAC |
| ScMMF1 (F) | AGCCTGTTCAAGGTTCTATCTCTGA |
| ScMMF1 (R) | ACATTCAAAGGTAAGGAAGCAACAC |
| ScOAC1 (F) | TGGTCAAAAATGATCTGATGAAAGA |
| ScOAC1 (R) | CGGTTACACCTTCGATTCTAACAGT |
| ScFUM1 (F) | TACTGATGCCGCATATTCATTTAGA |
| ScFUM1 (R) | CTTTTCAGTCAATACACCCAATTCC |
| ScCOX17 ePAT | GGCTTCGAAGTTCCAAGTGC |
| ScTOM70 ePAT | CGAACAGGGTTTAATGTAAACAG |
| ScOM14 ePAT | GGGTCTTTTGACGCTGGAC |
